# Genome-wide scan for runs of homozygosity identifies candidate genes in Wannan Black pigs

**DOI:** 10.5713/ab.20.0679

**Published:** 2021-03-10

**Authors:** Xudong Wu, Ren Zhou, Wei Zhang, Bangji Cao, Jing Xia, Caiyun Wang, Xiaodong Zhang, Mingxing Chu, Zongjun Yin, Yueyun Ding

**Affiliations:** 1 College of Animal Science and Technology, Anhui Agricultural University, Hefei, Anhui 230036, China; 2 Anhui Province Key Laboratory of Local Livestock and Poultry Genetic Resource Conservation and Bio-Breeding, Anhui Agricultural University, Hefei, 230036, China; 3 Key Laboratory of Pig Molecular Quantitative Genetics of Anhui Academy of Agricultural Sciences, Anhui Provincial Key Laboratory of Livestock and Poultry Product Safety Engineering, Institute of Animal Husbandry and Veterinary Medicine, Anhui Academy of Agricultural Sciences, Hefei, Anhui 230031, China; 4 Key Laboratory of Animal (Poultry) Genetics Breeding and Reproduction, Ministry of Agriculture and Rural Affairs, Institute of Animal Sciences, Chinese Academy of Agricultural Sciences, Beijing, 100193, China

**Keywords:** Inbreeding Coefficient, Native Breed, Pig Breeding, Runs of Homozygosity

## Abstract

**Objective:**

Runs of homozygosity (ROH) are contiguous lengths of homozygous genotypes that can reveal inbreeding levels, selection pressure, and mating schemes. In this study, ROHs were evaluated in Wannan Black pigs to assess the inbreeding levels and the genome regions with high ROH frequency.

**Methods:**

In a previous study, we obtained 501.52 GB of raw data from resequencing (10×) of the genome and identified 21,316,754 single-nucleotide variants in 20 Wannan Black pig samples. We investigated the number, length, and frequency of ROH using resequencing data to characterize the homozygosity in Wannan Black pigs and identified genomic regions with high ROH frequencies.

**Results:**

In this work, 1,813 ROHs (837 ROHs in 100 to 500 kb, 449 ROHs in 500 to 1,000 kb, 527 ROHs in >1,000 kb) were identified in all samples, and the average genomic inbreeding coefficient (F_ROH_) in Wannan Black pigs was 0.5234. Sixty-one regions on chromosomes 2, 3, 7, 8, 13, 15, and 16 harbored ROH islands. In total, 105 genes were identified in 42 ROH islands, among which some genes were related to production traits.

**Conclusion:**

This is the first study to identify ROH across the genome of Wannan Black pigs, the Chinese native breed of the Anhui province. Overall, Wannan Black pigs have high levels of inbreeding due to the influence of ancient and recent inbreeding due to the genome. These findings are a reliable resource for future studies and contribute to save and use the germplasm resources of Wannan Black pigs.

## INTRODUCTION

Pigs were among the first species that were domesticated. They provide an important meat resource and serve as a biomedical animal model for human [1]. Domestication of wild boars began approximately 10,000 years ago [2], and hundreds of pig breeds evolved worldwide due to natural selection and artificial selection pressure. For a long time, pig breeding was the main method of increasing the economic performance of pig production [3]. The fertility, growth speed, and meat quality of pigs is significantly improved over that of the wild boar.

Molecular genetics is key to understanding the biodiversity and evolutionary relationships, and breeding programs [4]. DNA technologies are improving continuously and being increasingly applied to investigate the genotype of animals subjected to breeding [5]. Compared with traditional selection, molecular-assisted selection has advantages of seed selection and shortened breeding period in pigs. However, increased inbreeding is an inevitable consequence of genetic selection in livestock populations [6]. Inbreeding induces impaired performance traits (inbreeding depression) and reduced genetic variation and therefore, is an important factor to consider in animal breeding practices [7–9]. Inbreeding is usually estimated from the pedigree information of the animal populations. Incomplete records and neglectful historical inbreeding leads to the high error rate estimation and underestimation of the true inbreeding levels [10]. With the reducing cost of resequencing, it is possible to use genome-wide single-nucleotide polymorphism (SNP) information to assess true population genomic inbreeding levels [11].

Runs of homozygosity (ROH) are contiguous lengths of homozygous genotypes that are present in an individual due to parents transmitting identical haplotypes to their offspring [12]. Longer haplotypes are inherited from recent common ancestors and shorter haplotypes are inherited from distant ancestors. ROHs are not randomly distributed across the genome [13], and the distribution of ROH islands across the genome shows that the genome was subjected to selective pressure [14]. Furthermore, ROH has been widely used as a predictor of whole genome inbreeding levels. Past studies have shown that genomic inbreeding coefficient (F_ROH_) is a more accurate surrogate of inbreeding than the inbreeding coefficient estimated from the pedigree [15].

To the best of our knowledge, numerous reports have revealed that ROH has been used to explore the selection signatures in sheep [16], horses [17], and Western pig breeds [18]. However, few studies have focused on ROH analysis in Chinese pig breeds. Wannan Black pig has been bred for more than 100 years in mountainous area of southern Anhui province. Wannan Black pig were historically bred in dark and damp environment and two important factors were chosen for their breeding: reproductive performance and adaptive capacity [19]. As a native Chinese breed endemic to the local agriculture which exhibits disease resistance, high prolificacy, and high fat deposition [20], Wannan Black pig makes an important contribution to the local commercial pork production. In our previous work, we identified several candidate genes that have mutations in Wannan Black pigs that are significantly associated with production traits [21–23]. In recent decades, Wannan Black pigs have been in various unfavorable predicaments, leading to their low population density and diversity, greatly influenced by commercial, lean pig genotypes. From 1982 to 2019, the numbers of Wannan black pig sows changed from 6,688 to 360. Importantly, the risk of high level of inbreeding increased in small populations where the choice of breeding mates is limited [24]. On the other hand, some inbreeding depression in fitness-related traits of Wannan Black pigs has already been reported (Ministry of Agriculture and Rural Affairs of the People’s Republic of China, http://www.moa.gov.cn/). In order to provide reference for rational and efficient utilization of Wannan black pig germplasm resources, the frequency, number, and F_ROH_ of ROHs in Wannan black pig population were detected.

## MATERIALS AND METHODS

### Sequencing, genetic diversity and genetic relationship analysis

Our research was approved by the Anhui Agricultural University Animal Ethics Committee under permission No. AHAU20140215. In this study, 20 ear tissues of Wannan Black pigs (10 females and 10 males) were collected from the Wannan Black pig conservation farm, Huangshan City, Anhui province, People’s Republic of China). In a previous work, these samples were re-sequenced on the Illumina HiSeq X Ten platform (Illumina, San Diego, CA, USA) at Novogene Biotech Co., Ltd. (Beijing, China) with an average depth of ten generations. The analytical procedures related to the resequencing were adapted from Zhang et al [25]. Based on sequencing data, 501.52 G of raw data of the Wannan Black pig genome were obtained, which have been submitted to the National Center for Biotechnology Information (NCBI) database under the accession number PRJNA524263. A total of 21,316,754 SNPs were identified. We used the PLINK’-genome’ command to analyze the genetic relationship between the 20 pigs, as described by Purrcell et al [26].

### Runs of homozygosity detection criteria

ROH were identified for each animal using PLINK v1.07 software [26]. The following criteria were chosen for ROH estimation [27–29]: i) the minimum length of the filter input regions was set to 1 Mb; ii) one heterozygous and five missing calls were allowed per window to account for genotyping error; iii) the minimum number of SNPs was set to 100; and iv) the minimum quality of bases in the filter input regions was set to 10.

### Inbreeding coefficient estimation

Based on ROH data, genomic inbreeding for each animal was estimated from ROH (F_ROH_) as the ratio of the total length of the genome covered by ROH to the total length of the genome covered by SNPs or sequences, which is given as follows:

$$F_{ROH}=\frac{L_{ROH}}{L_{genome}}$$

Where L_ROH_ is the total length of an individual’s ROH in the autosomal genome, and *Lgenome* is the length of the autosomal genome covered by the SNPs, which was 2,435 G in this study.

### Detection of common runs of homozygosity

To identify genomic regions with a high frequency of ROH, we take the population as a unit, in which the ROH ratio of SNP sites in ROH was counted for each SNP site. Manhattan map was drawn according to ROH ratio of each SNP site. The ROH ratio where the top 1% is located is taken as the threshold line of high frequency SNP, and ROH island is obtained according to the distribution of SNP sites exceeding the threshold in the genome [30].

The gene content of the ROH islands was annotated using the annotation database provided by NCBI (https://www.ncbi.nlm.nih.gov). To further analyze the functions of the identified genes, gene ontology (GO) and Kyoto encyclopedia of genes and genomes (KEGG) analyses were performed using Database for Annotation, Visualization and Integrated Discovery (DAVID, v. 6.8) [31]. An extensive accurate literature search was then performed.

## RESULTS

The results of the genetic relationship analysis of the 20 Wannan Black pigs (Supplementary Table S1) showed that the animals were not related, which was useful for elucidating the ROH information.

### Runs of homozygosity detection and inbreeding coefficient

**Figure 1:**
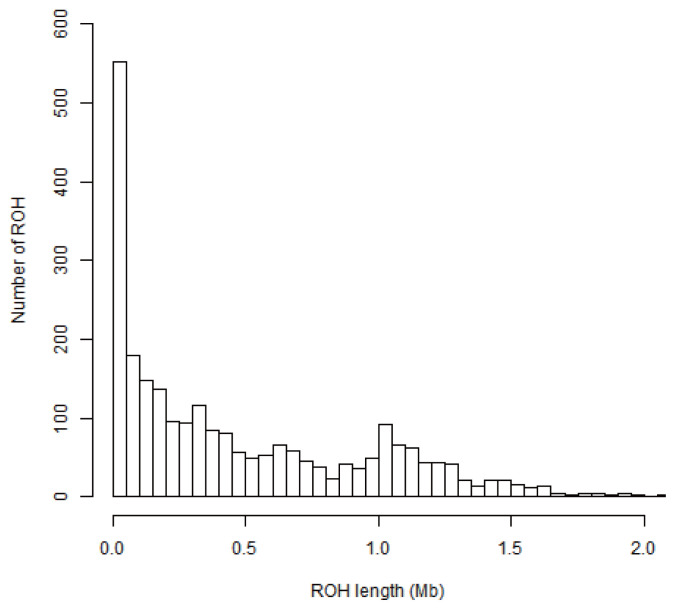
Distribution of the runs of homozygosity.

The descriptive statistics of ROH number and length by class are given in Table 1 and Figure 1. In total, 1,813 ROHs were identified in 20 Wannan Black pigs. Among the identified ROHs, 837 ranged between 100 to 500 kb, 449 between 500 to 1,000 kb, and 527 ROHs were >1,000 kb, with a mean ROH length of 0.69 MB. The longest segment was found in Sus Scrofa chromosome (SSC) 2, which was 3.42 Mb, and the shortest segment was 0.01 Mb. The total ROH number of Wannan Black pigs was composed mostly of a high number of shorter segments (100 to 500 kb and 500 to 1,000 kb) which accounted for 70.94% of all ROH detected. Although the number of ROHs of 100 to 500 kb was the largest, the proportion of the genome covered by them was relatively small compared to that of ROH segments longer than 1,000 kb.

**Figure 2:**
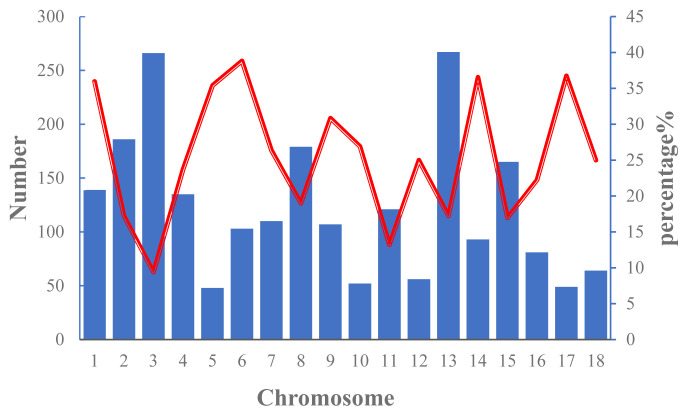
Number of runs of homozygosity (ROH) longer per chromosome (bars) and average percentage of each chromosome covered by ROH (red line).

The numbers of ROH on each chromosome and the percentage of chromosomes covered by ROH in Wannan Black pigs are shown in Figure 2. The results indicated that ROHs were distributed unevenly, with SSC13 and SSC5 showing the highest (n = 267) and lowest (n = 48) number of ROHs, respectively. The highest genome coverage by ROH was observed on SSC6 (38.84%), whereas the lowest coverage was on SSC3 (9.40%).

From the sequencing data, we inferred that 2,435,262,063 bp long autosomes were covered by SNPs and 1,274,547,767 bp long ROH were present on autosomes. Based on this information, the average FROH of Wannan Black pigs was 0.523.

### Identification of runs of homozygosity islands

**Figure 3:**
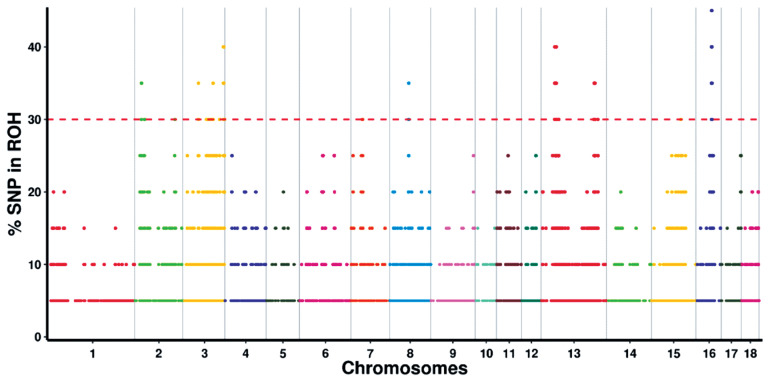
Manhattan plot of incidence of each single-nucleotide polymorphism (SNP) in the runs of homozygosity (ROH) across individuals. The red line represents the 30% threshold.

By determining the frequency of SNPs in ROH, we identified the genomic regions that were most commonly associated with ROHs in Wannan Black pigs. The results were plotted against the positions of the SNPs along the autosome. A total of 61 ROH islands were identified on SSC2, 3, 7, 8, 13, 15, and 16 (Figure 3; Supplementary Table S2). The longest ROH island was observed on SSC3, whereas the shortest one was observed on SSC13.

### Functional annotation of genes in the runs of homozygosity islands

Furthermore, the 61 ROH islands harbored 105 candidate genes, whereas 19 ROH islands did not harbor any genes (Supplementary Table S2). The function of the genes in the identified regions was analyzed (Supplementary Table S3, S4). Eight genes including eukaryotic translation initiation factor 1 (*EIF1*), nucleoporin 54 (*Nup54*), mitochondrial ribosomal protein S9 (*MRPS9*), and ribosomal protein S7 (*Rps7*) were related to signal translation processes, and four genes (four and a half LIM domains 2 [*FHL2*], leucine rich repeat containing 4C [*LRRC4C*], cytoplasmic polyadenylation element binding protein 4 ([*PEB4*], and EPH receptor A3 [*EPHA3*]) were involved in development. The remaining genes were involved in the immune system, nucleotide metabolism, amino acid metabolism, cellular community, and other important biological processes. Based on the results of the literature query, five genes were associated with economic traits in animals (Table 2).

## DISCUSSION

ROH are continuous homozygous segments of the DNA sequence in diploid genomes and are widely distributed in humans and livestock populations [32]. Inbreeding level has a significant influence on abundance, length, and number of ROH. In addition, genetic drift, natural and artificial selection, population bottlenecks among several other factors promote ROH buildup in a population [33]. However, ROH segments are not randomly distributed across the genome. In Chinese Merino sheep, *Ovies aries* chromosome (OAR) 21 and OAR3 exhibited the highest and lowest coverage across the chromosomes by ROH, respectively [34]. Xu et al [28] reported that in Jinhua pigs, the highest number of ROH per chromosome was on SSC6 with 1,672 segments, and the lowest was on SSC16 with 485 segments. In agreement with these findings, the SSC13 of Wannan Black pigs harbored the highest number of ROHs (n = 267), whereas SSC5 had the lowest number (n = 48).

The information regarding the abundance, length, and number of ROHs on the chromosome is valuable in exploring the demographic history of livestock species. With the availability of genomics tools, numerous polymorphisms can be synchronously genotyped across the genome and help to identify long stretches of homozygous genotypes [35]. In this work, the distribution and frequency of ROH in Wannan Black pig population were analyzed by resequencing (10×) data. Several studies have reported that long ROHs are associated with recent events of inbreeding, whereas short ROHs have been broken by repeated recombination and represent inbreeding that took place several generations ago [36,37]. A total of 1,813 ROHs were identified and short segments (lengths <1,000 kb) account for 70.93%. Wannan Black pig may therefore have undergone inbreeding in a distant generation, which concurs with what we know of the history of the Wannan Black pig.

Conventionally, coefficients of inbreeding were estimated from pedigree records. However, native breed conservation may suffer imperfections, such as the inaccurate pedigree records and chaotic consanguinity. Hence, ROH can be used as a predictor of whole genome inbreeding levels. FROH is defined as the proportion of the autosomal genome in ROH exceeding a specified length. McQuillan et al [38] first reported that FROH strongly correlates with the inbreeding coefficient of pedigrees in European populations. Our results showed that the average FROH in Wannan Black pigs was higher than that of other pig breeds (Złotnicka Spotted, Polish Landrace, 0.287, 0.171, respectively) [39]. Li et al [40] analyzed the genetic diversity of the same Wannan Black pig population (from the same conservation farm), and reported that these animals have more allele frequencies of microsatellite loci deviated from that expected under Hardy-Weinberg Equilibrium than introduced pig breeds (Duroc, Berkshire, Pietrain, Landrance, and Yorkshire) and other native pig breeds of Anhui province (Dinyuan pig, Wei pig, and Anqing six-end-white pig), revealing that Wannan Black pig has a higher inbreeding level than other pigs. With a small population size, inbreeding increases at a higher rate, leading to loss of alleles, which is impossible to counterbalance without migration [41]. Introducing new consanguinity, and increasing the effective population size of Wannan Black pig is necessary in the future.

Several candidate genes associated with production traits were identified in the ROH islands. Calmodulin-lysine N-methyltransferase (*CAMKMT*) is located on SSC3 of Wannan Black pig, which is significantly correlated with the number of embryonic cells and bone surface area [42]. The polymorphisms SNP07, SNP28, and SNP31 of *CAMKMT* polymorphisms are associated with sheep growth traits [43]. *CPEB4* is involved in bone, muscle, fat, and lung development, which is related to growth traits in Large White pig populations by genome wide association study [44]. The expression of collagen type III alpha 1 chain (*Col3a1*) was reported to affect intramuscular collagen, which is associated with meat quality in pigs [45]. Co-analysis of ROH and the signature selection identified GULP PTB domain containing engulfment adaptor 1 (*GULP1*) and kelch repeat and BTB domain containing 8 (*KBTBD8*) genes. *GULP1* is located on SSC15 and plays key roles in cancer suppression and immune responses [46], whereas *KBTBD8* is involved in cytoskeleton arrangement, regulation of cell morphology, and idiopathic short stature [47,48]. These two genes might be associated with the characteristics of Wannan Black pigs that adapt to natural and artificial selection. Therefore, elucidating the potential involvement of *GULP1* and *KBTBD8* in pig productive performance warrants further research.

## IMPLICATIONS

We identified runs of homozygosity s across the genome of the Wannan Black pig, the Chinese native breeds of the Anhui province, for the first time. The frequency, numbers, and FROH were obtained and reflected the inbreeding history in the Wannan Black pigs. Our results showed that ancient and recent inbreeding had an influence on the genome and revealed a high level of inbreeding in the existing population. Furthermore, several candidate genes associated with important biological processes were identified. In conclusion, our work will help to save and utilize the germplasm resource of Wannan Black pig.

## Figures and Tables

**Table 1:** Descriptive statistics of ROH number and length (in kb) by ROH length class

ROH length (kb)	ROH number	Percentage (%)	Mean length (Mb) (mean±SD)	Genome coverage (%)
100–500	837	46.17	0.27±0.11	9.16
500–1,000	449	24.77	0.72±0.14	13.35
>1,000	527	29.07	1.34±0.88	29.05
Total >100	1,813	100	0.69±8.81	51.56

ROH, runs of homozygosity.

**Table 2:** Candidate genes located in genomic regions with a high frequency of runs of homozygosity associated with economic traits

Chromosome	Location (bp)	Size (bp)	Gene	Gene function
3	95,854,107 – 95,918,246	64,139	calmodulin-lysine N-methyltransferase	Growth
15	92,482,944 – 93,357,026	874,082	GULP PTB domain containing engulfment adaptor 1	Immune responses
15	93,556,914 – 93,595,678	38,764	collagen type III alpha 1 chain	Meat quality
16	50,185,113 – 505,01,620	316,507	cytoplasmic polyadenylation element binding protein 4	Growth
13	48,486,919 – 48,499,992	13,073	kelch repeat and BTB domain containing 8	Growth
