# Supplementary material for: Genome-wide scan for runs of homozygosity identifies candidate genes in Wannan Black pigs
Source: Anim Biosci. 2021 Mar 10;34(12):1895–902. doi: 10.5713/ab.20.0679 (PMC8563231; doi:10.5713/ab.20.0679)
Supplement: Supplementary file 1 [file ab-20-0679-suppl.pdf]

S1 Genetic relationship analysis

Note:

Genome invokes an IBS/IBD computation,

FID1 Family ID for first sample

IID1 Individual ID for first sample

FID2 Family ID for second sample

IID3 Individual ID for second sample

RT Relationship type inferred from. fam/.ped file

EZ IBD sharing excepted value, based on just .fam/.ped relationship

Z0 P(IBD=0)

Z1 P(IBD=1)

Z2 P(IBD=2)

PI_HAT Protection IBD, i.e.P(IBD=2)＋0.5*P(IBD=1)

PHE Pairwise phenotypic code (1,0,-1=AA,AU, and UU pairs, respectively)

DST IBS distance,i.e.(IBS2＋0.5*IBS1)/(IBS0＋IBS1＋IBS2)

PPC IBS binomial test

RATIO HETHET :IBS0 SNP ratio (expected value 2)

FID1	IID1	FID2	IID2	RT	EZ	Z0	Z1	Z2	PI_HAT	PHE	DST	PPC	RATIO
S17	S17	WHJ1	WHJ1	UN	NA	1	0	0	0	-1	0.748	0	0.5679
S17	S17	WHJ11	WHJ11	UN	NA	1	0	0	0	-1	0.741	0	0.7774
S17	S17	WHJ12	WHJ12	UN	NA	1	0	0	0	-1	0.776	0	0.7249
S17	S17	WHJ16	WHJ16	UN	NA	1	0	0	0.125	-1	0.781	0	0.8463
S17	S17	WHJ18	WHJ18	UN	NA	1	0	0	0	-1	0.744	0	0.4641
S17	S17	WHJ19	WHJ19	UN	NA	1	0	0	0	-1	0.748	0	0.3863

S17	S17	WHJ20	WHJ20	UN	NA	1	0	0	0	-1	0.774	0	0.7863
S17	S17	WHJ21	WHJ21	UN	NA	1	0	0	0	-1	0.778	0	0.7014
S17	S17	WHJ22	WHJ22	UN	NA	1	0	0	0	-1	0.772	0	0.8952
S17	S17	WHJ23	WHJ23	UN	NA	1	0	0	0	-1	0.759	0	0.6673
S17	S17	WHJ24	WHJ24	UN	NA	1	0	0	0	-1	0.749	0	0.6916
S17	S17	WHJ27	WHJ27	UN	NA	1	0	0	0	-1	0.738	0	0.5148
S17	S17	WHJ28	WHJ28	UN	NA	1	0	0	0	-1	0.776	0	1.0204
S17	S17	WHJ29	WHJ29	UN	NA	1	0	0	0	-1	0.745	0	0.4984
S17	S17	WHJ3	WHJ3	UN	NA	1	0	0	0	-1	0.73	0	0.6323
S17	S17	WHJ4	WHJ4	UN	NA	1	0	0	0	-1	0.74	0	0.6594
S17	S17	WHJ5	WHJ5	UN	NA	1	0	0	0.303	-1	0.829	0	1.3911
S17	S17	WHJ6	WHJ6	UN	NA	1	0	0	0	-1	0.772	0	0.7568
S17	S17	WHJ8	WHJ8	UN	NA	1	0	0	0.129	-1	0.779	0	0.922
WHJ1	WHJ1	WHJ11	WHJ11	UN	NA	1	0	0	0	-1	0.756	0	0.6048
WHJ1	WHJ1	WHJ12	WHJ12	UN	NA	1	0	0	0	-1	0.763	0	0.4363
WHJ1	WHJ1	WHJ16	WHJ16	UN	NA	1	0	0	0	-1	0.753	0	0.5612
WHJ1	WHJ1	WHJ18	WHJ18	UN	NA	1	0	0	0	-1	0.772	0	0.3292
WHJ1	WHJ1	WHJ19	WHJ19	UN	NA	1	0	0	0	-1	0.776	0	0.2717
WHJ1	WHJ1	WHJ20	WHJ20	UN	NA	1	0	0	0	-1	0.748	0	0.4528
WHJ1	WHJ1	WHJ21	WHJ21	UN	NA	1	0	0	0	-1	0.76	0	0.3159
WHJ1	WHJ1	WHJ22	WHJ22	UN	NA	1	0	0	0	-1	0.741	0	0.4485
WHJ1	WHJ1	WHJ23	WHJ23	UN	NA	1	0	0	0	-1	0.765	0	0.5035
WHJ1	WHJ1	WHJ24	WHJ24	UN	NA	1	0	0	0	-1	0.75	0	0.4134
WHJ1	WHJ1	WHJ27	WHJ27	UN	NA	1	0	0	0	-1	0.768	0	0.4253
WHJ1	WHJ1	WHJ28	WHJ28	UN	NA	1	0	0	0	-1	0.759	0	0.5781
WHJ1	WHJ1	WHJ29	WHJ29	UN	NA	1	0	0	0	-1	0.747	0	0.3636

WHJ1	WHJ1	WHJ3	WHJ3	UN	NA	1	0	0	0	-1	0.757	0	0.4948
WHJ1	WHJ1	WHJ4	WHJ4	UN	NA	1	0	0	0	-1	0.763	0	0.4873
WHJ1	WHJ1	WHJ5	WHJ5	UN	NA	1	0	0	0	-1	0.742	0	0.512
WHJ1	WHJ1	WHJ6	WHJ6	UN	NA	1	0	0	0	-1	0.761	0	0.5045
WHJ1	WHJ1	WHJ8	WHJ8	UN	NA	1	0	0	0	-1	0.763	0	0.5596
WHJ11	WHJ11	WHJ12	WHJ12	UN	NA	1	0	0	0	-1	0.756	0	0.6472
WHJ11	WHJ11	WHJ16	WHJ16	UN	NA	1	0	0	0	-1	0.757	0	0.8066
WHJ11	WHJ11	WHJ18	WHJ18	UN	NA	1	0	0	0	-1	0.757	0	0.4895
WHJ11	WHJ11	WHJ19	WHJ19	UN	NA	1	0	0	0	-1	0.778	0	0.5065
WHJ11	WHJ11	WHJ20	WHJ20	UN	NA	1	0	0	0	-1	0.744	0	0.663
WHJ11	WHJ11	WHJ21	WHJ21	UN	NA	1	0	0	0	-1	0.741	0	0.519
WHJ11	WHJ11	WHJ22	WHJ22	UN	NA	1	0	0	0	-1	0.738	0	0.693
WHJ11	WHJ11	WHJ23	WHJ23	UN	NA	1	0	0	0	-1	0.782	0	0.7633
WHJ11	WHJ11	WHJ24	WHJ24	UN	NA	1	0	0	0	-1	0.771	0	0.7578
WHJ11	WHJ11	WHJ27	WHJ27	UN	NA	1	0	0	0	-1	0.772	0	0.6838
WHJ11	WHJ11	WHJ28	WHJ28	UN	NA	1	0	0	0	-1	0.738	0	0.7739
WHJ11	WHJ11	WHJ29	WHJ29	UN	NA	1	0	0	0	-1	0.791	0	0.6964
WHJ11	WHJ11	WHJ3	WHJ3	UN	NA	1	0	0	0.271	-1	0.82	0	1.1104
WHJ11	WHJ11	WHJ4	WHJ4	UN	NA	1	0	0	0.309	-1	0.829	0	1.3868
WHJ11	WHJ11	WHJ5	WHJ5	UN	NA	1	0	0	0	-1	0.737	0	0.6954
WHJ11	WHJ11	WHJ6	WHJ6	UN	NA	1	0	0	0	-1	0.769	0	0.7989
WHJ11	WHJ11	WHJ8	WHJ8	UN	NA	1	0	0	0	-1	0.746	0	0.8359
WHJ12	WHJ12	WHJ16	WHJ16	UN	NA	1	0	0	0	-1	0.779	0	0.7324
WHJ12	WHJ12	WHJ18	WHJ18	UN	NA	1	0	0	0	-1	0.755	0	0.3559
WHJ12	WHJ12	WHJ19	WHJ19	UN	NA	1	0	0	0	-1	0.796	0	0.4015
WHJ12	WHJ12	WHJ20	WHJ20	UN	NA	1	0	0	0	-1	0.748	0	0.5131

WHJ12	WHJ12	WHJ21	WHJ21	UN	NA	1	0	0	0	-1	0.746	0	0.367
WHJ12	WHJ12	WHJ22	WHJ22	UN	NA	1	0	0	0	-1	0.729	0	0.4976
WHJ12	WHJ12	WHJ23	WHJ23	UN	NA	1	0	0	0	-1	0.79	0	0.6738
WHJ12	WHJ12	WHJ24	WHJ24	UN	NA	1	0	0	0	-1	0.782	0	0.6254
WHJ12	WHJ12	WHJ27	WHJ27	UN	NA	1	0	0	0	-1	0.761	0	0.4347
WHJ12	WHJ12	WHJ28	WHJ28	UN	NA	1	0	0	0	-1	0.743	0	0.6006
WHJ12	WHJ12	WHJ29	WHJ29	UN	NA	1	0	0	0	-1	0.752	0	0.3962
WHJ12	WHJ12	WHJ3	WHJ3	UN	NA	1	0	0	0	-1	0.742	0	0.4718
WHJ12	WHJ12	WHJ4	WHJ4	UN	NA	1	0	0	0	-1	0.753	0	0.5382
WHJ12	WHJ12	WHJ5	WHJ5	UN	NA	1	0	0	0	-1	0.771	0	0.6968
WHJ12	WHJ12	WHJ6	WHJ6	UN	NA	1	0	0	0.318	-1	0.829	0	1.0105
WHJ12	WHJ12	WHJ8	WHJ8	UN	NA	1	0	0	0	-1	0.747	0	0.5742
WHJ16	WHJ16	WHJ18	WHJ18	UN	NA	1	0	0	0	-1	0.755	0	0.4813
WHJ16	WHJ16	WHJ19	WHJ19	UN	NA	1	0	0	0	-1	0.778	0	0.4782
WHJ16	WHJ16	WHJ20	WHJ20	UN	NA	1	0	0	0	-1	0.75	0	0.673
WHJ16	WHJ16	WHJ21	WHJ21	UN	NA	1	0	0	0	-1	0.746	0	0.5205
WHJ16	WHJ16	WHJ22	WHJ22	UN	NA	1	0	0	0	-1	0.736	0	0.6546
WHJ16	WHJ16	WHJ23	WHJ23	UN	NA	1	0	0	0	-1	0.788	0	0.7887
WHJ16	WHJ16	WHJ24	WHJ24	UN	NA	1	0	0	0	-1	0.769	0	0.7362
WHJ16	WHJ16	WHJ27	WHJ27	UN	NA	1	0	0	0	-1	0.794	0	0.7902
WHJ16	WHJ16	WHJ28	WHJ28	UN	NA	1	0	0	0	-1	0.752	0	0.8279
WHJ16	WHJ16	WHJ29	WHJ29	UN	NA	1	0	0	0	-1	0.766	0	0.6229
WHJ16	WHJ16	WHJ3	WHJ3	UN	NA	1	0	0	0	-1	0.751	0	0.6662
WHJ16	WHJ16	WHJ4	WHJ4	UN	NA	1	0	0	0	-1	0.754	0	0.7287
WHJ16	WHJ16	WHJ5	WHJ5	UN	NA	1	0	0	0.195	-1	0.802	0	1.054
WHJ16	WHJ16	WHJ6	WHJ6	UN	NA	1	0	0	0.19	-1	0.792	0	0.8799

WHJ16	WHJ16	WHJ8	WHJ8	UN	NA	1	0	0	0	-1	0.753	0	0.799
WHJ18	WHJ18	WHJ19	WHJ19	UN	NA	1	0	0	0	-1	0.774	0	0.2243
WHJ18	WHJ18	WHJ20	WHJ20	UN	NA	1	0	0	0	-1	0.753	0	0.4103
WHJ18	WHJ18	WHJ21	WHJ21	UN	NA	1	0	0	0	-1	0.773	0	0.2927
WHJ18	WHJ18	WHJ22	WHJ22	UN	NA	1	0	0	0	-1	0.748	0	0.4226
WHJ18	WHJ18	WHJ23	WHJ23	UN	NA	1	0	0	0	-1	0.767	0	0.4371
WHJ18	WHJ18	WHJ24	WHJ24	UN	NA	1	0	0	0	-1	0.748	0	0.3495
WHJ18	WHJ18	WHJ27	WHJ27	UN	NA	1	0	0	0	-1	0.782	0	0.3831
WHJ18	WHJ18	WHJ28	WHJ28	UN	NA	1	0	0	0	-1	0.766	0	0.5396
WHJ18	WHJ18	WHJ29	WHJ29	UN	NA	1	0	0	0	-1	0.762	0	0.312
WHJ18	WHJ18	WHJ3	WHJ3	UN	NA	1	0	0	0	-1	0.759	0	0.4445
WHJ18	WHJ18	WHJ4	WHJ4	UN	NA	1	0	0	0	-1	0.752	0	0.3878
WHJ18	WHJ18	WHJ5	WHJ5	UN	NA	1	0	0	0	-1	0.754	0	0.4498
WHJ18	WHJ18	WHJ6	WHJ6	UN	NA	1	0	0	0	-1	0.765	0	0.4336
WHJ18	WHJ18	WHJ8	WHJ8	UN	NA	1	0	0	0	-1	0.76	0	0.482
WHJ19	WHJ19	WHJ20	WHJ20	UN	NA	1	0	0	0	-1	0.75	0	0.322
WHJ19	WHJ19	WHJ21	WHJ21	UN	NA	1	0	0	0	-1	0.754	0	0.2262
WHJ19	WHJ19	WHJ22	WHJ22	UN	NA	1	0	0	0	-1	0.722	0	0.2938
WHJ19	WHJ19	WHJ23	WHJ23	UN	NA	1	0	0	0.42	-1	0.86	0	0.8668
WHJ19	WHJ19	WHJ24	WHJ24	UN	NA	1	0	0	0	-1	0.78	0	0.3577
WHJ19	WHJ19	WHJ27	WHJ27	UN	NA	1	0	0	0	-1	0.812	0	0.402
WHJ19	WHJ19	WHJ28	WHJ28	UN	NA	1	0	0	0	-1	0.741	0	0.3786
WHJ19	WHJ19	WHJ29	WHJ29	UN	NA	1	0	0	0	-1	0.8	0	0.3271
WHJ19	WHJ19	WHJ3	WHJ3	UN	NA	1	0	0	0	-1	0.769	0	0.3954
WHJ19	WHJ19	WHJ4	WHJ4	UN	NA	1	0	0	0	-1	0.771	0	0.3839
WHJ19	WHJ19	WHJ5	WHJ5	UN	NA	1	0	0	0	-1	0.749	0	0.3556

WHJ19	WHJ19	WHJ6	WHJ6	UN	NA	1	0	0	0	-1	0.807	0	0.4877
WHJ19	WHJ19	WHJ8	WHJ8	UN	NA	1	0	0	0	-1	0.76	0	0.4403
WHJ20	WHJ20	WHJ21	WHJ21	UN	NA	1	0	0	0	-1	0.765	0	0.5444
WHJ20	WHJ20	WHJ22	WHJ22	UN	NA	1	0	0	0	-1	0.77	0	0.7794
WHJ20	WHJ20	WHJ23	WHJ23	UN	NA	1	0	0	0	-1	0.751	0	0.5679
WHJ20	WHJ20	WHJ24	WHJ24	UN	NA	1	0	0	0	-1	0.75	0	0.599
WHJ20	WHJ20	WHJ27	WHJ27	UN	NA	1	0	0	0	-1	0.748	0	0.4869
WHJ20	WHJ20	WHJ28	WHJ28	UN	NA	1	0	0	0	-1	0.764	0	0.7829
WHJ20	WHJ20	WHJ29	WHJ29	UN	NA	1	0	0	0	-1	0.734	0	0.4354
WHJ20	WHJ20	WHJ3	WHJ3	UN	NA	1	0	0	0	-1	0.732	0	0.541
WHJ20	WHJ20	WHJ4	WHJ4	UN	NA	1	0	0	0	-1	0.747	0	0.5563
WHJ20	WHJ20	WHJ5	WHJ5	UN	NA	1	0	0	0	-1	0.782	0	0.8379
WHJ20	WHJ20	WHJ6	WHJ6	UN	NA	1	0	0	0	-1	0.756	0	0.602
WHJ20	WHJ20	WHJ8	WHJ8	UN	NA	1	0	0	0.159	-1	0.794	0	0.9664
WHJ21	WHJ21	WHJ22	WHJ22	UN	NA	1	0	0	0	-1	0.755	0	0.5378
WHJ21	WHJ21	WHJ23	WHJ23	UN	NA	1	0	0	0	-1	0.752	0	0.4638
WHJ21	WHJ21	WHJ24	WHJ24	UN	NA	1	0	0	0	-1	0.744	0	0.3891
WHJ21	WHJ21	WHJ27	WHJ27	UN	NA	1	0	0	0	-1	0.748	0	0.3092
WHJ21	WHJ21	WHJ28	WHJ28	UN	NA	1	0	0	0	-1	0.792	0	0.8549
WHJ21	WHJ21	WHJ29	WHJ29	UN	NA	1	0	0	0	-1	0.749	0	0.3203
WHJ21	WHJ21	WHJ3	WHJ3	UN	NA	1	0	0	0	-1	0.736	0	0.4001
WHJ21	WHJ21	WHJ4	WHJ4	UN	NA	1	0	0	0	-1	0.743	0	0.4282
WHJ21	WHJ21	WHJ5	WHJ5	UN	NA	1	0	0	0	-1	0.788	0	0.6774
WHJ21	WHJ21	WHJ6	WHJ6	UN	NA	1	0	0	0	-1	0.754	0	0.4451
WHJ21	WHJ21	WHJ8	WHJ8	UN	NA	1	0	0	0	-1	0.782	0	0.7554
WHJ22	WHJ22	WHJ23	WHJ23	UN	NA	1	0	0	0	-1	0.734	0	0.5329

WHJ22	WHJ22	WHJ24	WHJ24	UN	NA	1	0	0	0	-1	0.734	0	0.5734
WHJ22	WHJ22	WHJ27	WHJ27	UN	NA	1	0	0	0	-1	0.729	0	0.4856
WHJ22	WHJ22	WHJ28	WHJ28	UN	NA	1	0	0	0	-1	0.765	0	0.9267
WHJ22	WHJ22	WHJ29	WHJ29	UN	NA	1	0	0	0	-1	0.734	0	0.4536
WHJ22	WHJ22	WHJ3	WHJ3	UN	NA	1	0	0	0	-1	0.735	0	0.6223
WHJ22	WHJ22	WHJ4	WHJ4	UN	NA	1	0	0	0	-1	0.736	0	0.6103
WHJ22	WHJ22	WHJ5	WHJ5	UN	NA	1	0	0	0	-1	0.759	0	0.7542
WHJ22	WHJ22	WHJ6	WHJ6	UN	NA	1	0	0	0	-1	0.733	0	0.5733
WHJ22	WHJ22	WHJ8	WHJ8	UN	NA	1	0	0	0	-1	0.775	0	0.9532
WHJ23	WHJ23	WHJ24	WHJ24	UN	NA	1	0	0	0	-1	0.77	0	0.6086
WHJ23	WHJ23	WHJ27	WHJ27	UN	NA	1	0	0	0	-1	0.809	0	0.7068
WHJ23	WHJ23	WHJ28	WHJ28	UN	NA	1	0	0	0	-1	0.755	0	0.6992
WHJ23	WHJ23	WHJ29	WHJ29	UN	NA	1	0	0	0.357	-1	0.837	0	0.6504
WHJ23	WHJ23	WHJ3	WHJ3	UN	NA	1	0	0	0	-1	0.772	0	0.6861
WHJ23	WHJ23	WHJ4	WHJ4	UN	NA	1	0	0	0	-1	0.775	0	0.6977
WHJ23	WHJ23	WHJ5	WHJ5	UN	NA	1	0	0	0	-1	0.755	0	0.6446
WHJ23	WHJ23	WHJ6	WHJ6	UN	NA	1	0	0	0.219	-1	0.799	0	0.727
WHJ23	WHJ23	WHJ8	WHJ8	UN	NA	1	0	0	0	-1	0.764	0	0.6986
WHJ24	WHJ24	WHJ27	WHJ27	UN	NA	1	0	0	0	-1	0.761	0	0.5397
WHJ24	WHJ24	WHJ28	WHJ28	UN	NA	1	0	0	0	-1	0.737	0	0.6724
WHJ24	WHJ24	WHJ29	WHJ29	UN	NA	1	0	0	0	-1	0.778	0	0.5219
WHJ24	WHJ24	WHJ3	WHJ3	UN	NA	1	0	0	0	-1	0.754	0	0.5632
WHJ24	WHJ24	WHJ4	WHJ4	UN	NA	1	0	0	0	-1	0.763	0	0.6364
WHJ24	WHJ24	WHJ5	WHJ5	UN	NA	1	0	0	0	-1	0.758	0	0.7274
WHJ24	WHJ24	WHJ6	WHJ6	UN	NA	1	0	0	0	-1	0.784	0	0.7749
WHJ24	WHJ24	WHJ8	WHJ8	UN	NA	1	0	0	0	-1	0.745	0	0.6447

WHJ27	WHJ27	WHJ28	WHJ28	UN	NA	1	0	0	0	-1	0.75	0	0.6055
WHJ27	WHJ27	WHJ29	WHJ29	UN	NA	1	0	0	0	-1	0.78	0	0.5051
WHJ27	WHJ27	WHJ3	WHJ3	UN	NA	1	0	0	0	-1	0.765	0	0.5383
WHJ27	WHJ27	WHJ4	WHJ4	UN	NA	1	0	0	0	-1	0.775	0	0.6033
WHJ27	WHJ27	WHJ5	WHJ5	UN	NA	1	0	0	0	-1	0.75	0	0.555
WHJ27	WHJ27	WHJ6	WHJ6	UN	NA	1	0	0	0	-1	0.784	0	0.6722
WHJ27	WHJ27	WHJ8	WHJ8	UN	NA	1	0	0	0	-1	0.758	0	0.6232
WHJ28	WHJ28	WHJ29	WHJ29	UN	NA	1	0	0	0	-1	0.741	0	0.5488
WHJ28	WHJ28	WHJ3	WHJ3	UN	NA	1	0	0	0	-1	0.733	0	0.6688
WHJ28	WHJ28	WHJ4	WHJ4	UN	NA	1	0	0	0	-1	0.743	0	0.7057
WHJ28	WHJ28	WHJ5	WHJ5	UN	NA	1	0	0	0	-1	0.765	0	0.9061
WHJ28	WHJ28	WHJ6	WHJ6	UN	NA	1	0	0	0	-1	0.755	0	0.7945
WHJ28	WHJ28	WHJ8	WHJ8	UN	NA	1	0	0	0.146	-1	0.788	0	1.1459
WHJ29	WHJ29	WHJ3	WHJ3	UN	NA	1	0	0	0	-1	0.781	0	0.5381
WHJ29	WHJ29	WHJ4	WHJ4	UN	NA	1	0	0	0	-1	0.776	0	0.5632
WHJ29	WHJ29	WHJ5	WHJ5	UN	NA	1	0	0	0	-1	0.744	0	0.5149
WHJ29	WHJ29	WHJ6	WHJ6	UN	NA	1	0	0	0	-1	0.765	0	0.5443
WHJ29	WHJ29	WHJ8	WHJ8	UN	NA	1	0	0	0	-1	0.74	0	0.4766
WHJ3	WHJ3	WHJ4	WHJ4	UN	NA	1	0	0	0.308	-1	0.828	0	1.1247
WHJ3	WHJ3	WHJ5	WHJ5	UN	NA	1	0	0	0	-1	0.733	0	0.6432
WHJ3	WHJ3	WHJ6	WHJ6	UN	NA	1	0	0	0	-1	0.757	0	0.6113
WHJ3	WHJ3	WHJ8	WHJ8	UN	NA	1	0	0	0	-1	0.733	0	0.6716
WHJ4	WHJ4	WHJ5	WHJ5	UN	NA	1	0	0	0	-1	0.735	0	0.6046
WHJ4	WHJ4	WHJ6	WHJ6	UN	NA	1	0	0	0	-1	0.764	0	0.6456
WHJ4	WHJ4	WHJ8	WHJ8	UN	NA	1	0	0	0	-1	0.745	0	0.7403
WHJ5	WHJ5	WHJ6	WHJ6	UN	NA	1	0	0	0	-1	0.769	0	0.7563

WHJ5	WHJ5	WHJ8	WHJ8	UN	NA	1	0	0	0	-1	0.776	0	0.908
WHJ6	WHJ6	WHJ8	WHJ8	UN	NA	1	0	0	0	-1	0.754	0	0.6626

S2 Genomic regions with a high frequency of ROH

chr	start	end	snp_count	gene
15	92482944	93357026	3828	GULP1
3	128880521	128913961	483	RSAD2,CMPK2
13	43204298	43287703	527	
13	48118769	48119148	4	
16	49193152	49345636	970	DRD1
3	127865889	128552612	8165	
13	44113672	44392134	1870	CADPS
3	49273862	49424659	1682	FHL2
3	128552613	128553397	13	
16	49345637	49641222	1991	Gtf2a2
16	50185113	50501620	428	Pol,NSG2,C5orf47,CPEB4
8	61029341	61038882	12	
13	167542233	167573464	82	
13	169706304	169781185	498	CADM2
3	49521329	49544906	267	TGFBRAP1,GPR45
3	95854107	95918246	262	CAMKMT
3	128747884	128880520	1895	RNF144A,RSAD2
2	21274497	22083127	6743	LRRC4C
13	54838390	55877846	5522	Pdzrn3,CNTN3
13	169781186	170218962	1880	CADM2,CADM2
2	126947279	127203487	2815	CSNK1G3
7	35803690	36414177	7445	LRFN2,UNC5CL,TSPO2,APOBEC2,OARD1,NFYA,ADCY10
2	21076964	21274496	1796	

13	42784539	43204297	1827	
3	82603922	83352327	2089	FANCL
16	50539686	51016189	1390	BOD1,STC2
13	44927233	44987176	412	SYNPR
3	131013568	131837975	11577	Dcdc2c,ALLC,COLEC11,Rps7,RNASEH1,ADI1,TRAPPC12,EIPR1
3	94758375	95512404	4807	PRKCE,Nutf2,SRBD1,SIX2
13	46888780	47400284	3378	MAGI1,MAGI1
3	127839689	127840106	6	
3	79645919	79860589	853	HMGN1,B3GNT2,Nup54,COMMD1
13	47400285	47428359	154	MAGI1
3	95512405	95854106	2661	SIX3,CAMKMT
7	35543982	35749214	2242	LRFN2
3	115792887	116177602	2419	
3	88411027	89517161	5243	
8	60249516	61029340	2563	LPHN3,ADGRL3
13	166509127	167542232	6071	EPHA3
13	44392135	44392580	3	CADPS
3	74632702	75395375	5061	ETAA1
13	43939312	44113671	500	FEZF2,CADPS
13	43287704	43465154	1001	PTPRG
2	30821688	31804045	6401	RPL35A
3	128553398	128747883	2425	RNF144A
3	49692824	49697828	25	
13	178985579	179632840	4476	LIPI,RBM11,ABCC1,HSPA13,SAMSN1
8	60030724	60249515	810	
16	50501621	50539685	108	

16	49641223	50185112	2189	Gtf2a2,MSX2
13	47428360	48096740	4198	MAGI1,SLC25A26,LRIG1
16	49140044	49193151	155	TNPO1,DRD1
13	43465155	43939311	2701	PTPRG,C3orf14
16	51087289	51111241	127	NKX2-5
3	127840107	127865888	323	
16	51059490	51087288	166	NKX2-5
13	170218963	170440433	1471	
3	49544907	49692823	1319	MRPS9
13	48096741	48118768	145	
13	167573465	167749664	1042	
16	51016190	51059489	188	

S3 KEGG enrichment analysis

Pathway Name	Pathway ID	Pvalue_adjusted	Count
Ribosome	ssc03010	0.108	3
Sulfur relay system	ssc04122	0.32	1
RNA transport	ssc03013	0.328	2
Folate biosynthesis	ssc00790	0.328	1
Phototransduction	ssc04744	0.328	1
Axon guidance	ssc04360	0.328	2
SNARE interactions in vesicular transport	ssc04130	0.328	1
DNA replication	ssc03030	0.328	1
cAMP signaling pathway	ssc04024	0.328	2
Basal transcription factors	ssc03022	0.331	1
Cocaine addiction	ssc05030	0.334	1
Amphetamine addiction	ssc05031	0.363	1
Gap junction	ssc04540	0.363	1
Progesterone-mediated oocyte maturation	ssc04914	0.363	1
Morphine addiction	ssc05032	0.363	1
TGF-beta signaling pathway	ssc04350	0.363	1
Protein digestion and absorption	ssc04974	0.363	1
Circadian entrainment	ssc04713	0.363	1
Amoebiasis	ssc05146	0.363	1
AGE-RAGE signaling pathway in diabetic complications	ssc04933	0.363	1
Oocyte meiosis	ssc04114	0.369	1
Platelet activation	ssc04611	0.369	1

Dopaminergic synapse	ssc04728		0.369	1
Purine metabolism	ssc00230		0.369	1
Signaling pathways regulating pluripotency of stem cells	ssc04550		0.369	1
Cell adhesion molecules	ssc04514		0.369	1
Hippo signaling pathway	ssc04390		0.369	1
Influenza A	ssc05164		0.369	1
Alcoholism	ssc05034		0.369	1
Transcriptional misregulation in cancer	ssc05202		0.39	1
Viral carcinogenesis	ssc05203		0.401	1
Calcium signaling pathway	ssc04020		0.408	1
Parkinson disease	ssc05012		0.47	1
Neuroactive ligand-receptor interaction	ssc04080		0.572	1
Metabolic pathways	ssc01100	1.00e+00		1
Dorso-ventral axis formation	ssc04320	1.00e+00		1

S4 GO enrichment analysis

Molecular function

GO_Name	GO_ID	Pvalue_adjusted	Count
poly(A) RNA binding	GO:0044822	0.0461	9
transcription factor activity, transcription factor binding	GO:0000989	0.0461	6
transcription factor activity, protein binding	GO:0000988	0.0461	6
S-adenosyl-L-methionine transmembrane transporter activity	GO:0000095	0.0675	1
acireductone dioxygenase [iron(II)-requiring] activity	GO:0010309	0.0675	1
calcium-independent protein kinase C activity	GO:0004699	0.0675	1
rRNA (cytosine-N4-)-methyltransferase activity	GO:0071424	0.0675	1
bicarbonate binding	GO:0071890	0.0675	1
structural molecule activity	GO:0005198	0.0675	9
nucleocytoplasmic transporter activity	GO:0005487	0.0675	2
transcription cofactor activity	GO:0003712	0.07	6
transcriptional repressor activity, RNA polymerase II activating transcription factor binding	GO:0098811	0.07	2
structural constituent of ribosome	GO:0003735	0.07	6
transcription factor activity, RNA polymerase II core promoter proximal region sequence-specific binding	GO:0000982	0.07	4
rRNA (cytosine) methyltransferase activity	GO:0016434	0.0743	1
UMP kinase activity	GO:0033862	0.0743	1
phosphotyrosine residue binding	GO:0001784	0.077	2
regulatory region DNA binding	GO:0000975	0.0775	6
transcriptional activator activity, RNA polymerase II transcription factor binding	GO:0001190	0.0775	2
translation factor activity, RNA binding	GO:0008135	0.0775	3
protein phosphorylated amino acid binding	GO:0045309	0.0775	2
allantoicase activity	GO:0004037	0.0775	1
serum response element binding	GO:0010736	0.0775	1

Ran GTPase binding	GO:0008536	0.0809	2
4 iron, 4 sulfur cluster binding	GO:0051539	0.0828	2
ligase inhibitor activity	GO:0055104	0.0913	1
transcriptional activator activity, RNA polymerase II core promoter proximal region sequence-specific binding	GO:0001077	0.0956	3
transcriptional repressor activity, RNA polymerase II transcription factor binding	GO:0001191	0.0956	2
thymidylate kinase activity	GO:0004798	0.0956	1
ethanol binding	GO:0035276	0.0956	1
ubiquitin ligase inhibitor activity	GO:1990948	0.0956	1
ubiquitin-protein transferase activity	GO:0004842	0.103	5
GPI-linked ephrin receptor activity	GO:0005004	0.103	1
coenzyme transporter activity	GO:0051185	0.103	1
transcription corepressor activity	GO:0003714	0.103	3
ubiquitin-like protein transferase activity	GO:0019787	0.108	5
uridylate kinase activity	GO:0009041	0.108	1
SMAD binding	GO:0046332	0.108	2
transcription factor binding	GO:0008134	0.108	5
mRNA 3'-UTR binding	GO:0003730	0.108	2
cytidylate kinase activity	GO:0004127	0.108	1
RNA-DNA hybrid ribonuclease activity	GO:0004523	0.108	1
alcohol binding	GO:0043178	0.108	2
transcription regulatory region sequence-specific DNA binding	GO:0000976	0.108	6
RNA binding	GO:0003723	0.108	11
phosphoprotein binding	GO:0051219	0.111	2
RNA polymerase II transcription cofactor activity	GO:0001104	0.111	2
G-protein coupled photoreceptor activity	GO:0008020	0.111	1
photoreceptor activity	GO:0009881	0.12	1

core promoter sequence-specific DNA binding	GO:0001046	0.125	2
glucocorticoid receptor binding	GO:0035259	0.126	1
iron-sulfur cluster binding	GO:0051536	0.126	2
metal cluster binding	GO:0051540	0.126	2
14-3-3 protein binding	GO:0071889	0.131	1
platelet-derived growth factor binding	GO:0048407	0.137	1
translation repressor activity, nucleic acid binding	GO:0000900	0.137	1
protein homodimerization activity	GO:0042803	0.138	5
core promoter proximal region DNA binding	GO:0001159	0.142	3
protein dimerization activity	GO:0046983	0.144	7
transcription coactivator activity	GO:0003713	0.163	3
retinoid X receptor binding	GO:0046965	0.163	1
transcription factor activity, RNA polymerase II transcription factor binding	GO:0001076	0.163	2
transcription regulatory region DNA binding	GO:0044212	0.163	6
mRNA binding	GO:0003729	0.163	3
nucleic acid binding transcription factor activity	GO:0001071	0.163	6
regulatory region nucleic acid binding	GO:0001067	0.163	6
RNA polymerase II regulatory region sequence-specific DNA binding	GO:0000977	0.163	5
sequence-specific double-stranded DNA binding	GO:1990837	0.163	5
RNA polymerase II regulatory region DNA binding	GO:0001012	0.163	5
mRNA 5'-UTR binding	GO:0048027	0.164	1
cofactor transporter activity	GO:0051184	0.164	1
core promoter binding	GO:0001047	0.164	2
identical protein binding	GO:0042802	0.164	7
phosphatidic acid binding	GO:0070300	0.164	1
growth factor binding	GO:0019838	0.164	2

translation regulator activity, nucleic acid binding	GO:0090079	0.177	1
fibroblast growth factor binding	GO:0017134	0.177	1
ephrin receptor activity	GO:0005003	0.177	1
sequence-specific DNA binding	GO:0043565	0.177	7
dopamine neurotransmitter receptor activity	GO:0004952	0.177	1
potassium ion binding	GO:0030955	0.177	1
phosphatidylinositol-3,4-bisphosphate binding	GO:0043325	0.177	1
RNA polymerase II transcription factor activity, sequence-specific DNA binding	GO:0000981	0.182	5
receptor activator activity	GO:0030546	0.182	1
nucleoside diphosphate kinase activity	GO:0004550	0.182	1
TBP-class protein binding	GO:0017025	0.182	1
ligand-dependent nuclear receptor binding	GO:0016922	0.182	1
translation repressor activity	GO:0030371	0.182	1
nucleotide kinase activity	GO:0019201	0.182	1
hydrolase activity, acting on carbon-nitrogen (but not peptide) bonds, in linear amidines	GO:0016813	0.184	1
rRNA methyltransferase activity	GO:0008649	0.184	1
double-stranded DNA binding	GO:0003690	0.184	5
protein kinase C activity	GO:0004697	0.191	1
alkali metal ion binding	GO:0031420	0.191	1
phosphatidylinositol-3,5-bisphosphate binding	GO:0080025	0.198	1
protein serine/threonine kinase activator activity	GO:0043539	0.198	1
estrogen receptor binding	GO:0030331	0.199	1
transcriptional activator activity, RNA polymerase II transcription regulatory region sequence-specific binding	GO:0001228	0.199	3
retinoic acid receptor binding	GO:0042974	0.199	1
phosphatidylinositol-3,4,5-trisphosphate binding	GO:0005547	0.199	1
adenylate cyclase activity	GO:0004016	0.199	1

nucleic acid binding	GO:0003676	0.201	20
RNA polymerase II repressing transcription factor binding	GO:0001103	0.201	1
alpha-actinin binding	GO:0051393	0.213	1
RNA polymerase II transcription coactivator activity	GO:0001105	0.213	1
protein binding	GO:0005515	0.216	25
actin monomer binding	GO:0003785	0.224	1
ubiquitin conjugating enzyme binding	GO:0031624	0.227	1
transcriptional repressor activity, RNA polymerase II transcription regulatory region sequence-specific binding	GO:0001227	0.231	2
nuclear localization sequence binding	GO:0008139	0.233	1
protein heterodimerization activity	GO:0046982	0.235	3
actinin binding	GO:0042805	0.235	1
sulfur compound transmembrane transporter activity	GO:1901682	0.235	1
scaffold protein binding	GO:0097110	0.235	1
ubiquitin-like protein conjugating enzyme binding	GO:0044390	0.237	1
cholesterol binding	GO:0015485	0.238	1
endoribonuclease activity, producing 5'-phosphomonoesters	GO:0016891	0.238	1
oxidoreductase activity, acting on single donors with incorporation of molecular oxygen, incorporation of two atoms of oxygen	GO:0016702	0.24	1
protein kinase regulator activity	GO:0019887	0.244	2
RNA polymerase II core promoter proximal region sequence-specific DNA binding	GO:0000978	0.245	3
protein self-association	GO:0043621	0.246	1
oxidoreductase activity, acting on single donors with incorporation of molecular oxygen	GO:0016701	0.246	1
delayed rectifier potassium channel activity	GO:0005251	0.248	1
cyclase activity	GO:0009975	0.25	1
core promoter proximal region sequence-specific DNA binding	GO:0000987	0.261	3
structural constituent of nuclear pore	GO:0017056	0.267	1

sterol binding	GO:0032934	0.27	1
kinase regulator activity	GO:0019207	0.277	2
phosphorus-oxygen lyase activity	GO:0016849	0.286	1
transforming growth factor beta receptor binding	GO:0005160	0.29	1
signal sequence binding	GO:0005048	0.29	1
phosphatidylinositol-4,5-bisphosphate binding	GO:0005546	0.29	1
metal ion binding	GO:0046872	0.29	17
translation regulator activity	GO:0045182	0.29	1
protein complex scaffold activity	GO:0032947	0.29	1
RNA polymerase II core promoter sequence-specific DNA binding	GO:0000979	0.29	1
transcription factor activity, RNA polymerase II distal enhancer sequence-specific binding	GO:0003705	0.29	1
SNAP receptor activity	GO:0005484	0.29	1
steroid hormone receptor binding	GO:0035258	0.291	1
enzyme binding	GO:0019899	0.291	8
repressing transcription factor binding	GO:0070491	0.294	1
endonuclease activity, active with either ribo- or deoxyribonucleic acids and producing 5'-phosphomonoesters	GO:0016893	0.295	1
binding	GO:0005488	0.295	51
tRNA binding	GO:0000049	0.302	1
ubiquitin protein ligase activity	GO:0061630	0.302	2
ubiquitin-like protein ligase activity	GO:0061659	0.307	2
cation binding	GO:0043169	0.309	17
phosphotransferase activity, phosphate group as acceptor	GO:0016776	0.311	1
endoribonuclease activity	GO:0004521	0.319	1
extracellular matrix structural constituent	GO:0005201	0.324	1
ribosome binding	GO:0043022	0.325	1
magnesium ion binding	GO:0000287	0.329	2

nucleobase-containing compound kinase activity	GO:0019205	0.34	1
RNA methyltransferase activity	GO:0008173	0.341	1
transcriptional repressor activity, RNA polymerase II core promoter proximal region sequence-specific binding	GO:0001078	0.342	1
protein kinase inhibitor activity	GO:0004860	0.346	1
kinase inhibitor activity	GO:0019210	0.357	1
phosphatidylinositol bisphosphate binding	GO:1902936	0.36	1
steroid binding	GO:0005496	0.362	1
transcription factor activity, sequence-specific DNA binding	GO:0003700	0.362	6
copper ion binding	GO:0005507	0.363	1
protein kinase activator activity	GO:0030295	0.363	1
ion channel binding	GO:0044325	0.371	1
translation initiation factor activity	GO:0003743	0.371	1
oxidoreductase activity, acting on the CH-CH group of donors	GO:0016627	0.371	1
kinase activator activity	GO:0019209	0.372	1
peptide transporter activity	GO:0015197	0.373	1
enhancer sequence-specific DNA binding	GO:0001158	0.373	1
histone deacetylase binding	GO:0042826	0.377	1
dioxygenase activity	GO:0051213	0.377	1
cell adhesion molecule binding	GO:0050839	0.378	2
protein transporter activity	GO:0008565	0.378	1
enhancer binding	GO:0035326	0.386	1
protease binding	GO:0002020	0.4	1
protein complex binding	GO:0032403	0.406	3
receptor binding	GO:0005102	0.409	6
integrin binding	GO:0005178	0.409	1
transmembrane receptor protein tyrosine kinase activity	GO:0004714	0.446	1

phospholipase activity	GO:0004620	0.458	1
RNA polymerase II transcription factor binding	GO:0001085	0.458	1
nuclear hormone receptor binding	GO:0035257	0.458	1
voltage-gated potassium channel activity	GO:0005249	0.462	1
ribonuclease activity	GO:0004540	0.462	1
macromolecular complex binding	GO:0044877	0.462	5
protein C-terminus binding	GO:0008022	0.462	1
heparin binding	GO:0008201	0.462	1
N-methyltransferase activity	GO:0008170	0.464	1
ribonucleoprotein complex binding	GO:0043021	0.464	1
phosphatidylinositol phosphate binding	GO:1901981	0.474	1
organic cyclic compound binding	GO:0097159	0.474	26
enzyme inhibitor activity	GO:0004857	0.474	2
hormone activity	GO:0005179	0.489	1
transmembrane receptor protein kinase activity	GO:0019199	0.503	1
Ras GTPase binding	GO:0017016	0.507	2
small GTPase binding	GO:0031267	0.515	2
DNA binding	GO:0003677	0.526	8
carboxylic ester hydrolase activity	GO:0052689	0.526	1
potassium channel activity	GO:0005267	0.526	1
hormone receptor binding	GO:0051427	0.526	1
transition metal ion binding	GO:0046914	0.526	6
lipase activity	GO:0016298	0.526	1
receptor activity	GO:0004872	0.526	6
endonuclease activity	GO:0004519	0.528	1
transmembrane receptor activity	GO:0099600	0.538	5

heterocyclic compound binding	GO:1901363	0.544	25
hydrolase activity, acting on carbon-nitrogen (but not peptide) bonds	GO:0016810	0.544	1
neurotransmitter receptor activity	GO:0030594	0.556	1
sulfur compound binding	GO:1901681	0.557	1
metalloendopeptidase activity	GO:0004222	0.56	1
enzyme regulator activity	GO:0030234	0.561	4
glycosaminoglycan binding	GO:0005539	0.573	1
protein tyrosine phosphatase activity	GO:0004725	0.578	1
transporter activity	GO:0005215	0.588	6
S-adenosylmethionine-dependent methyltransferase activity	GO:0008757	0.591	1
receptor regulator activity	GO:0030545	0.606	1
potassium ion transmembrane transporter activity	GO:0015079	0.607	1
GTPase binding	GO:0051020	0.608	2
peptide binding	GO:0042277	0.614	1
voltage-gated cation channel activity	GO:0022843	0.617	1
enzyme activator activity	GO:0008047	0.632	2
protein tyrosine kinase activity	GO:0004713	0.636	1
heme binding	GO:0020037	0.659	1
amide binding	GO:0033218	0.659	1
lyase activity	GO:0016829	0.659	1
zinc ion binding	GO:0008270	0.667	4
tetrapyrrole binding	GO:0046906	0.676	1
phosphatidylinositol binding	GO:0035091	0.679	1
nuclease activity	GO:0004518	0.681	1
carbohydrate binding	GO:0030246	0.683	1
voltage-gated ion channel activity	GO:0005244	0.683	1

voltage-gated channel activity	GO:0022832	0.683	1
iron ion binding	GO:0005506	0.683	1
transferase activity	GO:0016740	0.685	10
metallopeptidase activity	GO:0008237	0.698	1
endopeptidase activity	GO:0004175	0.698	2
lipid binding	GO:0008289	0.698	2
phosphoprotein phosphatase activity	GO:0004721	0.698	1
methyltransferase activity	GO:0008168	0.7	1
signal transducer activity	GO:0004871	0.7	5
protein serine/threonine kinase activity	GO:0004674	0.708	2
cytokine receptor binding	GO:0005126	0.708	1
transferase activity, transferring one-carbon groups	GO:0016741	0.708	1
hydrolase activity, acting on ester bonds	GO:0016788	0.714	3
kinase activity	GO:0016301	0.738	4
protein kinase activity	GO:0004672	0.767	3
ion binding	GO:0043167	0.783	21
phosphatase activity	GO:0016791	0.816	1
cation channel activity	GO:0005261	0.832	1
purine ribonucleoside binding	GO:0032550	0.832	4
purine nucleoside binding	GO:0001883	0.832	4
ribonucleoside binding	GO:0032549	0.832	4
phospholipid binding	GO:0005543	0.832	1
nucleoside binding	GO:0001882	0.832	4
transferase activity, transferring phosphorus-containing groups	GO:0016772	0.833	4
molecular function regulator	GO:0098772	0.849	4
gated channel activity	GO:0022836	0.849	1

phosphotransferase activity, alcohol group as acceptor	GO:0016773	0.849	3
peptidase activity, acting on L-amino acid peptides	GO:0070011	0.861	2
substrate-specific transporter activity	GO:0022892	0.863	2
monovalent inorganic cation transmembrane transporter activity	GO:0015077	0.863	1
chromatin binding	GO:0003682	0.863	1
peptidase activity	GO:0008233	0.863	2
phosphoric ester hydrolase activity	GO:0042578	0.901	1
actin binding	GO:0003779	0.901	1
metal ion transmembrane transporter activity	GO:0046873	0.915	1
ion channel activity	GO:0005216	0.915	1
substrate-specific channel activity	GO:0022838	0.919	1
cytoskeletal protein binding	GO:0008092	0.919	2
oxidoreductase activity	GO:0016491	0.919	2
passive transmembrane transporter activity	GO:0022803	0.919	1
channel activity	GO:0015267	0.919	1
G-protein coupled receptor activity	GO:0004930	0.943	4
catalytic activity	GO:0003824	0.962	21
inorganic cation transmembrane transporter activity	GO:0022890	0.965	1
substrate-specific transmembrane transporter activity	GO:0022891	0.965	1
transmembrane signaling receptor activity	GO:0004888	0.966	5
ATP binding	GO:0005524	0.969	4
adenyl ribonucleotide binding	GO:0032559	0.969	4
molecular transducer activity	GO:0060089	0.969	6
adenyl nucleotide binding	GO:0030554	0.969	4
cation transmembrane transporter activity	GO:0008324	0.973	1
signaling receptor activity	GO:0038023	0.973	5

anion binding	GO:0043168	0.974	7
calcium ion binding	GO:0005509	0.974	1
transmembrane transporter activity	GO:0022857	0.974	2
carbohydrate derivative binding	GO:0097367	0.974	5
hydrolase activity	GO:0016787	0.974	6
purine ribonucleoside triphosphate binding	GO:0035639	0.974	4
small molecule binding	GO:0036094	0.974	6
purine ribonucleotide binding	GO:0032555	0.974	4
purine nucleotide binding	GO:0017076	0.974	4
ion transmembrane transporter activity	GO:0015075	0.974	1
nucleoside phosphate binding	GO:1901265	0.974	5
nucleotide binding	GO:0000166	0.974	5
ribonucleotide binding	GO:0032553	0.974	4

Cell component

**GO_Name**	**GO_ID**	**Pvalue_adjusted**	**Count**
membrane-bounded organelle	GO:0043227	0.0109	48
small ribosomal subunit	GO:0015935	0.0109	5
intracellular membrane-bounded organelle	GO:0043231	0.0109	45
endomembrane system	GO:0012505	0.0109	20
cell junction	GO:0030054	0.0204	9
cytosolic small ribosomal subunit	GO:0022627	0.0206	4
protein complex	GO:0043234	0.0206	19
adherens junction	GO:0005912	0.0206	6
nucleus	GO:0005634	0.0206	32
anchoring junction	GO:0070161	0.0206	6

endoplasmic reticulum	GO:0005783	0.0237	10
ribosomal subunit	GO:0044391	0.0237	6
intracellular organelle	GO:0043229	0.0237	48
organelle	GO:0043226	0.0237	50
intracellular ribonucleoprotein complex	GO:0030529	0.0237	7
nuclear part	GO:0044428	0.0237	21
organelle part	GO:0044422	0.0241	34
photoreceptor inner segment membrane	GO:0060342	0.0241	1
cytosolic ribosome	GO:0022626	0.0369	5
collagen type III trimer	GO:0005586	0.0433	1
nuclear lumen	GO:0031981	0.0435	18
intracellular organelle lumen	GO:0070013	0.0435	19
organelle lumen	GO:0043233	0.0435	19
membrane-enclosed lumen	GO:0031974	0.0435	19
intracellular organelle part	GO:0044446	0.0467	32
molybdopterin synthase complex	GO:0019008	0.0486	1
ribosome	GO:0005840	0.0486	6
cytoplasm	GO:0005737	0.056	39
membrane-bounded vesicle	GO:0031988	0.0606	11
focal adhesion	GO:0005925	0.0606	4
ubiquitin ligase complex	GO:0000151	0.0606	4
cell-substrate adherens junction	GO:0005924	0.0606	4
nucleoplasm	GO:0005654	0.0606	14
extracellular region part	GO:0044421	0.0606	15
cell-substrate junction	GO:0030055	0.0606	4
cytoplasmic part	GO:0044444	0.0739	31

intracellular part	GO:0044424	0.0756	51
Cul3-RING ubiquitin ligase complex	GO:0031463	0.0756	2
cullin-RING ubiquitin ligase complex	GO:0031461	0.0779	3
early endosome	GO:0005769	0.0779	3
extracellular membrane-bounded organelle	GO:0065010	0.0795	9
cytosolic part	GO:0044445	0.0795	5
extracellular region	GO:0005576	0.0795	16
intrinsic component of synaptic vesicle membrane	GO:0098563	0.0795	1
photoreceptor outer segment membrane	GO:0042622	0.0795	1
intracellular	GO:0005622	0.0795	52
host	GO:0018995	0.0795	2
host cell	GO:0043657	0.0795	2
collagen trimer	GO:0005581	0.0795	2
other organism part	GO:0044217	0.0795	2
other organism cell	GO:0044216	0.0795	2
other organism	GO:0044215	0.0795	2
nuclear envelope	GO:0005635	0.0795	4
CORVET complex	GO:0033263	0.0795	1
vesicle	GO:0031982	0.0802	13
extracellular exosome	GO:0070062	0.0811	9
messenger ribonucleoprotein complex	GO:1990124	0.0814	1
extracellular vesicle	GO:1903561	0.0814	9
extracellular organelle	GO:0043230	0.0814	9
integral component of synaptic vesicle membrane	GO:0030285	0.0843	1
transcription factor TFIIA complex	GO:0005672	0.0843	1
proteinaceous extracellular matrix	GO:0005578	0.0848	3

banded collagen fibril	GO:0098643	0.087	1
fibrillar collagen trimer	GO:0005583	0.087	1
macromolecular complex	GO:0032991	0.0931	25
nuclear pore central transport channel	GO:0044613	0.104	1
extracellular matrix	GO:0031012	0.112	4
Cul2-RING ubiquitin ligase complex	GO:0031462	0.113	1
complex of collagen trimers	GO:0098644	0.139	1
Fanconi anaemia nuclear complex	GO:0043240	0.139	1
ribonucleoprotein complex	GO:1990904	0.145	7
cell part	GO:0044464	0.148	60
cell	GO:0005623	0.172	60
RNA polymerase II transcription factor complex	GO:0090575	0.188	2
exosome (RNase complex)	GO:0000178	0.19	1
photoreceptor inner segment	GO:0001917	0.193	1
ciliary membrane	GO:0060170	0.206	1
mitochondrial small ribosomal subunit	GO:0005763	0.211	1
organellar small ribosomal subunit	GO:0000314	0.211	1
perinuclear region of cytoplasm	GO:0048471	0.228	3
nuclear transcription factor complex	GO:0044798	0.24	2
cell-cell junction	GO:0005911	0.246	3
cytosol	GO:0005829	0.264	12
transcription factor TFIID complex	GO:0005669	0.275	1
90S preribosome	GO:0030686	0.275	1
pore complex	GO:0046930	0.283	1
nuclear membrane	GO:0031965	0.283	2
transferase complex	GO:1990234	0.283	5

small-subunit processome	GO:0032040	0.29	1
exocytic vesicle membrane	GO:0099501	0.29	1
neuromuscular junction	GO:0031594	0.301	1
nuclear replication fork	GO:0043596	0.301	1
synaptic vesicle membrane	GO:0030672	0.301	1
photoreceptor outer segment	GO:0001750	0.306	1
envelope	GO:0031975	0.307	5
organelle envelope	GO:0031967	0.307	5
intracellular non-membrane-bounded organelle	GO:0043232	0.307	16
non-membrane-bounded organelle	GO:0043228	0.307	16
SNARE complex	GO:0031201	0.317	1
histone deacetylase complex	GO:0000118	0.325	1
vesicle membrane	GO:0012506	0.329	2
lipid particle	GO:0005811	0.329	1
organelle membrane	GO:0031090	0.338	8
nonmotile primary cilium	GO:0031513	0.368	1
synapse	GO:0045202	0.374	4
extracellular space	GO:0005615	0.374	6
nuclear periphery	GO:0034399	0.374	1
transcriptional repressor complex	GO:0017053	0.374	1
cell-cell adherens junction	GO:0005913	0.381	1
replication fork	GO:0005657	0.388	1
Golgi apparatus	GO:0005794	0.388	5
endosome	GO:0005768	0.388	3
voltage-gated potassium channel complex	GO:0008076	0.388	1
endoplasmic reticulum subcompartment	GO:0098827	0.388	2

presynapse	GO:0098793	0.388	2
extracellular matrix component	GO:0044420	0.388	1
organellar ribosome	GO:0000313	0.388	1
mitochondrial ribosome	GO:0005761	0.388	1
transport vesicle membrane	GO:0030658	0.388	1
potassium channel complex	GO:0034705	0.388	1
synapse part	GO:0044456	0.388	3
nuclear pore	GO:0005643	0.388	1
primary cilium	GO:0072372	0.388	1
catalytic complex	GO:1902494	0.402	6
bicellular tight junction	GO:0005923	0.402	1
occluding junction	GO:0070160	0.402	1
centriole	GO:0005814	0.402	1
motile cilium	GO:0031514	0.411	1
preribosome	GO:0030684	0.411	1
asymmetric synapse	GO:0032279	0.434	1
neuron to neuron synapse	GO:0098984	0.434	1
kinetochore	GO:0000776	0.434	1
postsynaptic specialization	GO:0099572	0.438	1
apical junction complex	GO:0043296	0.439	1
transcription factor complex	GO:0005667	0.441	2
DNA-directed RNA polymerase II, holoenzyme	GO:0016591	0.447	1
recycling endosome	GO:0055037	0.452	1
synaptic vesicle	GO:0008021	0.476	1
exocytic vesicle	GO:0070382	0.477	1
microtubule organizing center part	GO:0044450	0.486	1

postsynaptic density	GO:0014069	0.5	1
cytoplasmic vesicle part	GO:0044433	0.503	2
neuron part	GO:0097458	0.508	4
cilium	GO:0005929	0.515	2
chromosome, centromeric region	GO:0000775	0.524	1
cell projection membrane	GO:0031253	0.525	1
nuclear DNA-directed RNA polymerase complex	GO:0055029	0.525	1
DNA-directed RNA polymerase complex	GO:0000428	0.525	1
RNA polymerase complex	GO:0030880	0.525	1
intracellular vesicle	GO:0097708	0.531	4
nucleolus	GO:0005730	0.573	3
cytoplasmic vesicle	GO:0031410	0.583	4
nuclear chromosome part	GO:0044454	0.597	2
chromosomal part	GO:0044427	0.597	3
centrosome	GO:0005813	0.597	2
endoplasmic reticulum membrane	GO:0005789	0.605	2
endosomal part	GO:0044440	0.605	1
cytosolic large ribosomal subunit	GO:0022625	0.605	1
nuclear chromosome	GO:0000228	0.607	2
cation channel complex	GO:0034703	0.607	1
nuclear outer membrane-endoplasmic reticulum membrane network	GO:0042175	0.607	2
bounding membrane of organelle	GO:0098588	0.611	4
transport vesicle	GO:0030133	0.616	1
mitochondrial matrix	GO:0005759	0.627	1
cytoplasmic vesicle membrane	GO:0030659	0.627	1
chromosome	GO:0005694	0.628	3

integral component of organelle membrane	GO:0031301	0.63	1
intrinsic component of organelle membrane	GO:0031300	0.641	1
endoplasmic reticulum part	GO:0044432	0.641	2
nuclear speck	GO:0016607	0.642	1
dendrite	GO:0030425	0.645	1
chromosomal region	GO:0098687	0.645	1
mitochondrion	GO:0005739	0.645	5
organelle subcompartment	GO:0031984	0.645	2
cell surface	GO:0009986	0.65	2
large ribosomal subunit	GO:0015934	0.653	1
microtubule organizing center	GO:0005815	0.675	2
nucleoplasm part	GO:0044451	0.675	3
postsynapse	GO:0098794	0.689	1
ion channel complex	GO:0034702	0.689	1
secretory vesicle	GO:0099503	0.703	1
ciliary part	GO:0044441	0.704	1
nuclear chromatin	GO:0000790	0.708	1
transferase complex, transferring phosphorus-containing groups	GO:0061695	0.708	1
microtubule cytoskeleton	GO:0015630	0.708	3
transmembrane transporter complex	GO:1902495	0.71	1
transporter complex	GO:1990351	0.713	1
somatodendritic compartment	GO:0036477	0.728	1
integral component of plasma membrane	GO:0005887	0.747	4
cell projection	GO:0042995	0.75	3
mitochondrial part	GO:0044429	0.75	2
membrane	GO:0016020	0.758	33

intrinsic component of plasma membrane	GO:0031226	0.767	4
mitochondrial inner membrane	GO:0005743	0.767	1
cytoplasmic, membrane-bounded vesicle	GO:0016023	0.782	1
membrane protein complex	GO:0098796	0.793	3
organelle inner membrane	GO:0019866	0.798	1
actin cytoskeleton	GO:0015629	0.817	1
chromatin	GO:0000785	0.822	1
nuclear body	GO:0016604	0.84	1
membrane region	GO:0098589	0.852	1
mitochondrial membrane	GO:0031966	0.855	1
cell projection part	GO:0044463	0.862	1
plasma membrane protein complex	GO:0098797	0.862	1
mitochondrial envelope	GO:0005740	0.869	1
cytoskeleton	GO:0005856	0.869	4
plasma membrane region	GO:0098590	0.87	1
neuron projection	GO:0043005	0.883	1
whole membrane	GO:0098805	0.883	2
plasma membrane part	GO:0044459	0.89	5
cytoskeletal part	GO:0044430	0.959	2
integral component of membrane	GO:0016021	0.977	19
intrinsic component of membrane	GO:0031224	0.977	19
plasma membrane	GO:0005886	0.977	10
cell periphery	GO:0071944	0.98	10
membrane part	GO:0044425	0.989	20

Biological process

GO_Name	GO_ID	Pvalue_adjusted	Count
single-organism process	GO:0044699	9.94E-06	54
single-organism cellular process	GO:0044763	0.000186	48
single-organism developmental process	GO:0044767	0.00617	24
single-multicellular organism process	GO:0044707	0.00966	27
embryonic digestive tract morphogenesis	GO:0048557	0.00966	3
rRNA processing	GO:0006364	0.0111	6
bundle of His development	GO:0003166	0.0111	2
rRNA metabolic process	GO:0016072	0.0164	6
His-Purkinje system development	GO:0003164	0.0181	2
embryonic digestive tract development	GO:0048566	0.0194	3
ventricular septum morphogenesis	GO:0060412	0.0194	3
negative regulation of macromolecule metabolic process	GO:0010605	0.0194	17
nervous system development	GO:0007399	0.0194	15
digestive tract development	GO:0048565	0.0204	4
negative regulation of transcription, DNA-templated	GO:0045892	0.0204	11
negative regulation of cellular macromolecule biosynthetic process	GO:2000113	0.0204	12
negative regulation of gene expression	GO:0010629	0.0204	13
digestive system development	GO:0055123	0.0204	4
negative regulation of nucleic acid-templated transcription	GO:1903507	0.0206	11
cardiac chamber morphogenesis	GO:0003206	0.0206	4
cellular response to acid chemical	GO:0071229	0.0206	4
negative regulation of RNA biosynthetic process	GO:1902679	0.0206	11
negative regulation of macromolecule biosynthetic process	GO:0010558	0.0214	12
digestive tract morphogenesis	GO:0048546	0.023	3
protein ubiquitination involved in ubiquitin-dependent protein catabolic process	GO:0042787	0.023	4

heart trabecula formation	GO:0060347	0.023	2
negative regulation of cellular metabolic process	GO:0031324	0.023	16
negative regulation of RNA metabolic process	GO:0051253	0.023	11
negative regulation of metabolic process	GO:0009892	0.023	17
positive regulation of nervous system development	GO:0051962	0.023	6
regulation of nervous system development	GO:0051960	0.023	8
negative regulation of cellular biosynthetic process	GO:0031327	0.023	12
translation	GO:0006412	0.023	10
cardiac ventricle formation	GO:0003211	0.0232	2
negative regulation of biosynthetic process	GO:0009890	0.0232	12
peptide biosynthetic process	GO:0043043	0.025	10
cardiac chamber development	GO:0003205	0.025	4
ventricular septum development	GO:0003281	0.025	3
cardiac septum morphogenesis	GO:0060411	0.025	3
cardiac chamber formation	GO:0003207	0.025	2
ventricular cardiac muscle cell development	GO:0055015	0.025	2
anatomical structure morphogenesis	GO:0009653	0.025	16
cellular biosynthetic process	GO:0044249	0.0256	31
cardiac conduction system development	GO:0003161	0.0266	2
ribosome biogenesis	GO:0042254	0.0288	6
embryonic organ morphogenesis	GO:0048562	0.0296	5
negative regulation of transcription from RNA polymerase II promoter	GO:0000122	0.0296	8
negative regulation of nucleobase-containing compound metabolic process	GO:0045934	0.0296	11
organonitrogen compound biosynthetic process	GO:1901566	0.0296	15
nitrogen compound metabolic process	GO:0006807	0.0297	47
system development	GO:0048731	0.0299	21

regulation of neurogenesis	GO:0050767	0.0299	7
ncRNA processing	GO:0034470	0.0299	6
response to acid chemical	GO:0001101	0.0299	4
amide biosynthetic process	GO:0043604	0.0299	10
cellular amide metabolic process	GO:0043603	0.0315	11
biosynthetic process	GO:0009058	0.0334	31
regulation of multicellular organismal process	GO:0051239	0.0344	15
neurogenesis	GO:0022008	0.0344	11
ventricular cardiac muscle cell differentiation	GO:0055012	0.0344	2
multicellular organism development	GO:0007275	0.0344	22
positive regulation of neurogenesis	GO:0050769	0.0344	5
metanephros development	GO:0001656	0.0361	3
peptide metabolic process	GO:0006518	0.0362	10
negative regulation of biological process	GO:0048519	0.0362	23
regulation of neuron differentiation	GO:0045664	0.0362	6
NLS-bearing protein import into nucleus	GO:0006607	0.0367	2
protein targeting to nucleus	GO:0044744	0.0373	4
trabecula formation	GO:0060343	0.0383	2
cardiac septum development	GO:0003279	0.0383	3
negative regulation of neurogenesis	GO:0050768	0.041	4
cell differentiation involved in metanephros development	GO:0072202	0.0415	2
tube development	GO:0035295	0.0415	7
negative regulation of nitrogen compound metabolic process	GO:0051172	0.0415	12
protein ubiquitination	GO:0016567	0.0415	8
cell development	GO:0048468	0.0421	13
organic substance biosynthetic process	GO:1901576	0.0425	30

organic substance metabolic process	GO:0071704	0.0438	50
negative regulation of B cell activation	GO:0050869	0.0438	2
negative regulation of gliogenesis	GO:0014014	0.0438	2
negative regulation of nervous system development	GO:0051961	0.0438	4
S-adenosyl-L-methionine transport	GO:0015805	0.0438	1
positive regulation of cellular glucuronidation	GO:2001031	0.0438	1
light absorption	GO:0016037	0.0438	1
positive regulation of hippo signaling	GO:0035332	0.0438	1
S-adenosyl-L-methionine transmembrane transport	GO:1901962	0.0438	1
endoplasmic reticulum membrane fusion	GO:0016320	0.0438	1
absorption of visible light	GO:0016038	0.0438	1
heart trabecula morphogenesis	GO:0061384	0.0438	2
muscle tissue development	GO:0060537	0.0438	5
negative regulation of cellular process	GO:0048523	0.0438	21
heart morphogenesis	GO:0003007	0.0438	4
generation of neurons	GO:0048699	0.0438	10
mesenchyme development	GO:0060485	0.0498	4
protein modification by small protein conjugation	GO:0032446	0.0514	8
cell differentiation	GO:0030154	0.0547	18
regulation of developmental process	GO:0050793	0.0551	13
leukocyte activation	GO:0045321	0.0551	7
tissue development	GO:0009888	0.0551	11
organonitrogen compound metabolic process	GO:1901564	0.0551	33
positive regulation of neuron differentiation	GO:0045666	0.0551	4
negative regulation of lymphocyte activation	GO:0051250	0.0551	3
cellular nitrogen compound metabolic process	GO:0034641	0.0566	32

embryonic morphogenesis	GO:0048598	0.0573	6
protein import into nucleus	GO:0006606	0.0573	4
cardiac ventricle development	GO:0003231	0.0573	3
cellular developmental process	GO:0048869	0.0573	19
regulation of cell cycle process	GO:0010564	0.0579	6
anatomical structure development	GO:0048856	0.0593	23
regulation of cell development	GO:0060284	0.0593	7
primary metabolic process	GO:0044238	0.0603	48
neuron differentiation	GO:0030182	0.0603	9
positive regulation of cell development	GO:0010720	0.0603	5
negative regulation of cell development	GO:0010721	0.0603	4
developmental process	GO:0032502	0.0603	24
protein import into nucleus, translocation	GO:0000060	0.0603	2
ribosomal small subunit biogenesis	GO:0042274	0.0603	3
ncRNA metabolic process	GO:0034660	0.0603	6
cardiac pacemaker cell fate commitment	GO:0060927	0.0603	1
macula densa development	GO:0072024	0.0603	1
atrioventricular node cell fate commitment	GO:0060929	0.0603	1
metanephric macula densa development	GO:0072227	0.0603	1
juxtaglomerular apparatus development	GO:0072051	0.0603	1
closure of optic fissure	GO:0061386	0.0603	1
positive regulation of lens epithelial cell proliferation	GO:2001111	0.0603	1
regulation of cellular glucuronidation	GO:2001029	0.0603	1
lens epithelial cell proliferation	GO:0097166	0.0603	1
Purkinje myocyte differentiation	GO:0003168	0.0603	1
regulation of lens epithelial cell proliferation	GO:2001109	0.0603	1

metanephric juxtaglomerular apparatus development	GO:0072206	0.0603	1
embryonic organ development	GO:0048568	0.0608	5
cellular nitrogen compound biosynthetic process	GO:0044271	0.0651	25
negative regulation of developmental process	GO:0051093	0.0659	7
negative regulation of cell differentiation	GO:0045596	0.0677	6
regulation of cellular component biogenesis	GO:0044087	0.0678	7
ribonucleoprotein complex biogenesis	GO:0022613	0.0678	6
negative regulation of DNA binding	GO:0043392	0.0678	2
negative regulation of leukocyte activation	GO:0002695	0.0678	3
trabecula morphogenesis	GO:0061383	0.0716	2
cellular component morphogenesis	GO:0032989	0.0716	9
cellular metabolic process	GO:0044237	0.0716	47
regulation of multicellular organismal development	GO:2000026	0.0716	10
axonogenesis	GO:0007409	0.0716	5
positive regulation of cellular component biogenesis	GO:0044089	0.0716	5
protein complex subunit organization	GO:0071822	0.0716	8
lymphocyte activation	GO:0046649	0.0716	6
cellular macromolecule metabolic process	GO:0044260	0.0716	39
metanephric thick ascending limb development	GO:0072233	0.0716	1
TRAM-dependent toll-like receptor signaling pathway	GO:0035668	0.0716	1
thick ascending limb development	GO:0072023	0.0716	1
TRAM-dependent toll-like receptor 4 signaling pathway	GO:0035669	0.0716	1
allantoin catabolic process	GO:0000256	0.0716	1
ovarian follicle rupture	GO:0001543	0.0716	1
DCT cell differentiation	GO:0072069	0.0716	1
atrioventricular node cell differentiation	GO:0060922	0.0716	1

positive regulation of toll-like receptor 7 signaling pathway	GO:0034157	0.0716	1
regulation of toll-like receptor 7 signaling pathway	GO:0034155	0.0716	1
cardiac septum cell differentiation	GO:0003292	0.0716	1
metanephric DCT cell differentiation	GO:0072240	0.0716	1
atrioventricular node cell development	GO:0060928	0.0716	1
His-Purkinje system cell differentiation	GO:0060932	0.0716	1
proepicardium development	GO:0003342	0.0716	1
septum transversum development	GO:0003343	0.0716	1
condensed mesenchymal cell proliferation	GO:0072137	0.0716	1
cell activation	GO:0001775	0.0717	7
heterocycle metabolic process	GO:0046483	0.073	28
stem cell differentiation	GO:0048863	0.0752	4
negative regulation of cell activation	GO:0050866	0.076	3
heart development	GO:0007507	0.0763	5
tube morphogenesis	GO:0035239	0.0764	5
regulation of metal ion transport	GO:0010959	0.0792	4
cellular response to amino acid stimulus	GO:0071230	0.0793	2
cardiac muscle cell development	GO:0055013	0.0802	2
animal organ morphogenesis	GO:0009887	0.0802	7
negative regulation of multicellular organismal process	GO:0051241	0.0802	7
axon development	GO:0061564	0.0802	5
cellular macromolecule biosynthetic process	GO:0034645	0.0802	24
cytoplasmic translation	GO:0002181	0.0802	3
cardiac cell development	GO:0055006	0.0802	2
cell differentiation involved in kidney development	GO:0061005	0.0802	2
protein import	GO:0017038	0.0802	4

cellular amide catabolic process	GO:0043605	0.0802	1
pulmonary myocardium development	GO:0003350	0.0802	1
positive regulation of chondrocyte proliferation	GO:1902732	0.0802	1
positive regulation of voltage-gated calcium channel activity	GO:1901387	0.0802	1
dUDP metabolic process	GO:0046077	0.0802	1
optic cup morphogenesis involved in camera-type eye development	GO:0002072	0.0802	1
allantoin metabolic process	GO:0000255	0.0802	1
negative regulation of B cell differentiation	GO:0045578	0.0802	1
protein targeting to nuclear inner membrane	GO:0036228	0.0802	1
atrioventricular node development	GO:0003162	0.0802	1
soft palate development	GO:0060023	0.0802	1
Purkinje myocyte development	GO:0003165	0.0802	1
septum secundum development	GO:0003285	0.0802	1
dUDP biosynthetic process	GO:0006227	0.0802	1
positive regulation of cell proliferation	GO:0008284	0.0802	6
protein modification by small protein conjugation or removal	GO:0070647	0.0819	8
ephrin receptor signaling pathway	GO:0048013	0.0824	2
cellular component biogenesis	GO:0044085	0.0839	15
ventricular cardiac muscle tissue development	GO:0003229	0.0839	2
epithelial tube morphogenesis	GO:0060562	0.0853	4
protein localization to nucleus	GO:0034504	0.0853	4
nucleobase-containing compound metabolic process	GO:0006139	0.0853	27
osteoblast differentiation	GO:0001649	0.0853	3
ossification	GO:0001503	0.0853	4
organic cyclic compound metabolic process	GO:1901360	0.0853	28
macromolecule metabolic process	GO:0043170	0.0853	42

mesenchymal cell differentiation	GO:0048762	0.0853	3
metanephric distal convoluted tubule development	GO:0072221	0.0853	1
distal convoluted tubule development	GO:0072025	0.0853	1
dTTP metabolic process	GO:0046075	0.0853	1
pyrimidine deoxyribonucleoside triphosphate biosynthetic process	GO:0009212	0.0853	1
aorta smooth muscle tissue morphogenesis	GO:0060414	0.0853	1
deoxyribonucleoside diphosphate biosynthetic process	GO:0009189	0.0853	1
dTDP metabolic process	GO:0046072	0.0853	1
embryonic heart tube left/right pattern formation	GO:0060971	0.0853	1
atrial cardiac muscle cell development	GO:0055014	0.0853	1
dTTP biosynthetic process	GO:0006235	0.0853	1
ventricular cardiac myofibril assembly	GO:0055005	0.0853	1
noradrenergic neuron differentiation	GO:0003357	0.0853	1
atrial cardiac muscle cell differentiation	GO:0055011	0.0853	1
pyrimidine deoxyribonucleoside diphosphate metabolic process	GO:0009196	0.0853	1
pyrimidine deoxyribonucleoside diphosphate biosynthetic process	GO:0009197	0.0853	1
dTDP biosynthetic process	GO:0006233	0.0853	1
pyrimidine nucleoside diphosphate biosynthetic process	GO:0009139	0.0853	1
positive regulation of translation in response to endoplasmic reticulum stress	GO:0036493	0.0853	1
mesenchymal stem cell proliferation	GO:0097168	0.0853	1
DNA replication, removal of RNA primer	GO:0043137	0.0853	1
DNA replication, Okazaki fragment processing	GO:0033567	0.0853	1
response to amino acid	GO:0043200	0.0856	2
macromolecule biosynthetic process	GO:0009059	0.0873	24
positive regulation of cellular process	GO:0048522	0.0883	21
maturation of SSU-rRNA from tricistronic rRNA transcript (SSU-rRNA, 5.8S rRNA, LSU-rRNA)	GO:0000462	0.0887	2

positive regulation of cell differentiation	GO:0045597	0.0887	6
striated muscle tissue development	GO:0014706	0.0887	4
neural crest cell development	GO:0014032	0.0887	2
cardiac muscle tissue development	GO:0048738	0.0887	3
circadian regulation of gene expression	GO:0032922	0.0887	2
animal organ development	GO:0048513	0.0887	14
cell morphogenesis	GO:0000902	0.0887	8
cell morphogenesis involved in differentiation	GO:0000904	0.0887	6
regulation of cell differentiation	GO:0045595	0.0887	9
cellular component organization or biogenesis	GO:0071840	0.0887	27
embryo development	GO:0009790	0.0887	7
negative regulation of immune system process	GO:0002683	0.0887	4
regulation of ossification	GO:0030278	0.0887	3
regulation of calcium ion transport	GO:0051924	0.0887	3
positive regulation of intrinsic apoptotic signaling pathway	GO:2001244	0.0887	2
extracellular matrix organization	GO:0030198	0.0887	5
purine nucleoside transmembrane transport	GO:0015860	0.0887	1
metanephric ascending thin limb development	GO:0072218	0.0887	1
ascending thin limb development	GO:0072021	0.0887	1
hard palate development	GO:0060022	0.0887	1
cardiac pacemaker cell development	GO:0060926	0.0887	1
positive regulation of intrinsic apoptotic signaling pathway by p53 class mediator	GO:1902255	0.0887	1
positive regulation of translation in response to stress	GO:0032056	0.0887	1
coenzyme transport	GO:0051182	0.0887	1
otolith morphogenesis	GO:0032474	0.0887	1
extracellular fibril organization	GO:0043206	0.0887	1

positive regulation of toll-like receptor 9 signaling pathway	GO:0034165	0.0887	1
regulation of kinetochore assembly	GO:0090234	0.0887	1
negative regulation of PERK-mediated unfolded protein response	GO:1903898	0.0887	1
right ventricular cardiac muscle tissue morphogenesis	GO:0003221	0.0887	1
negative regulation of glial cell proliferation	GO:0060253	0.0887	1
regulation of gliogenesis	GO:0014013	0.0896	2
outflow tract morphogenesis	GO:0003151	0.0896	2
epithelium development	GO:0060429	0.0896	7
cellular protein metabolic process	GO:0044267	0.0897	26
cell morphogenesis involved in neuron differentiation	GO:0048667	0.0914	5
cellular response to chemical stimulus	GO:0070887	0.0914	13
cellular response to oxygen-containing compound	GO:1901701	0.0926	6
cellular response to alcohol	GO:0097306	0.0935	2
extracellular structure organization	GO:0043062	0.0935	5
regulation of ion transport	GO:0043269	0.0935	5
cellular aromatic compound metabolic process	GO:0006725	0.0935	27
obsolete death	GO:0016265	0.0935	7
neural crest cell differentiation	GO:0014033	0.0935	2
negative regulation of protein modification process	GO:0031400	0.0935	5
deoxyribonucleoside triphosphate biosynthetic process	GO:0009202	0.0935	1
apoptotic process involved in heart morphogenesis	GO:0003278	0.0935	1
ribosomal protein import into nucleus	GO:0006610	0.0935	1
mesenchymal cell differentiation involved in renal system development	GO:2001012	0.0935	1
pyrimidine nucleoside diphosphate metabolic process	GO:0009138	0.0935	1
mesenchymal stem cell maintenance involved in nephron morphogenesis	GO:0072038	0.0935	1
cardiac pacemaker cell differentiation	GO:0060920	0.0935	1

negative regulation of endoplasmic reticulum stress-induced eIF2 alpha phosphorylation	GO:1903912	0.0935	1
negative regulation of transcription from RNA polymerase II promoter in response to endoplasmic reticulum stress	GO:1990441	0.0935	1
mesenchymal cell differentiation involved in kidney development	GO:0072161	0.0935	1
regulation of centromere complex assembly	GO:0090230	0.0935	1
enucleate erythrocyte differentiation	GO:0043353	0.0935	1
endodermal digestive tract morphogenesis	GO:0061031	0.0935	1
positive regulation of neuroepithelial cell differentiation	GO:1902913	0.0935	1
stem cell development	GO:0048864	0.0942	3
muscle tissue morphogenesis	GO:0060415	0.0948	2
positive regulation of cellular component organization	GO:0051130	0.0971	7
neuroepithelial cell differentiation	GO:0060563	0.0972	2
cardiac ventricle morphogenesis	GO:0003208	0.0972	2
regulation of translational initiation	GO:0006446	0.0972	2
regulation of sodium ion transport	GO:0002028	0.0972	2
regulation of lymphocyte activation	GO:0051249	0.098	4
negative regulation of developmental growth	GO:0048640	0.098	2
positive regulation of cell cycle process	GO:0090068	0.098	3
regulation of protein ubiquitination	GO:0031396	0.098	3
single organism signaling	GO:0044700	0.0992	21
positive regulation of T-helper 2 cell cytokine production	GO:2000553	0.0992	1
metanephric loop of Henle development	GO:0072236	0.0992	1
embryonic camera-type eye formation	GO:0060900	0.0992	1
positive regulation of melanocyte differentiation	GO:0045636	0.0992	1
cornea development in camera-type eye	GO:0061303	0.0992	1
epithelial cell differentiation involved in mammary gland alveolus development	GO:0061030	0.0992	1
negative regulation of collateral sprouting	GO:0048671	0.0992	1

metanephric distal tubule development	GO:0072235	0.0992	1
cellular response to ethanol	GO:0071361	0.0992	1
positive regulation of cardioblast differentiation	GO:0051891	0.0992	1
telencephalon development	GO:0021537	0.0992	3
glial cell development	GO:0021782	0.0994	2
cellular response to organic substance	GO:0071310	0.0994	11
muscle organ morphogenesis	GO:0048644	0.103	2
tissue morphogenesis	GO:0048729	0.104	5
transcription initiation from RNA polymerase II promoter	GO:0006367	0.104	2
regulation of cation transmembrane transport	GO:1904062	0.104	3
positive regulation of multicellular organismal process	GO:0051240	0.104	8
maturation of SSU-rRNA	GO:0030490	0.104	2
positive regulation of developmental process	GO:0051094	0.104	7
negative regulation of protein ubiquitination	GO:0031397	0.104	2
neuron projection morphogenesis	GO:0048812	0.104	5
positive regulation of cytoskeleton organization	GO:0051495	0.104	3
astral microtubule organization	GO:0030953	0.104	1
regulation of translation in response to endoplasmic reticulum stress	GO:0036490	0.104	1
ovulation from ovarian follicle	GO:0001542	0.104	1
regulation of translation initiation in response to endoplasmic reticulum stress	GO:0036491	0.104	1
deoxyribonucleoside diphosphate metabolic process	GO:0009186	0.104	1
regulation of T-helper 2 cell cytokine production	GO:2000551	0.104	1
regulation of hippo signaling	GO:0035330	0.104	1
interkinetic nuclear migration	GO:0022027	0.104	1
eiF2alpha phosphorylation in response to endoplasmic reticulum stress	GO:0036492	0.104	1
positive regulation of mucus secretion	GO:0070257	0.104	1

positive regulation of transcription via serum response element binding	GO:0010735	0.104	1
anatomical structure formation involved in morphogenesis	GO:0048646	0.106	7
positive regulation of hormone secretion	GO:0046887	0.106	2
neuron projection development	GO:0031175	0.107	6
positive regulation of synaptic transmission, GABAergic	GO:0032230	0.11	1
toll-like receptor 7 signaling pathway	GO:0034154	0.11	1
regulation of toll-like receptor 9 signaling pathway	GO:0034163	0.11	1
regulation of melanocyte differentiation	GO:0045634	0.11	1
protein localization to nuclear envelope	GO:0090435	0.11	1
otolith development	GO:0048840	0.11	1
molybdopterin cofactor biosynthetic process	GO:0032324	0.11	1
purine-containing compound transmembrane transport	GO:0072530	0.11	1
L-methionine salvage from methylthioadenosine	GO:0019509	0.11	1
endosomal vesicle fusion	GO:0034058	0.11	1
cardiac muscle cell fate commitment	GO:0060923	0.11	1
protein metabolic process	GO:0019538	0.11	28
regulation of protein modification by small protein conjugation or removal	GO:1903320	0.11	3
regulation of ion transmembrane transport	GO:0034765	0.11	4
chordate embryonic development	GO:0043009	0.113	5
negative regulation of protein modification by small protein conjugation or removal	GO:1903321	0.113	2
endoplasmic reticulum unfolded protein response	GO:0030968	0.113	2
nephron tubule development	GO:0072080	0.113	2
cardiac muscle cell differentiation	GO:0055007	0.113	2
nuclear transport	GO:0051169	0.114	4
embryo development ending in birth or egg hatching	GO:0009792	0.114	5
negative regulation of ubiquitin protein ligase activity	GO:1904667	0.114	1

pyrimidine deoxyribonucleoside triphosphate metabolic process	GO:0009211	0.114	1
L-methionine salvage	GO:0071267	0.114	1
positive regulation of pigment cell differentiation	GO:0050942	0.114	1
negative regulation of sodium ion transmembrane transport	GO:1902306	0.114	1
positive regulation of fibroblast migration	GO:0010763	0.114	1
membranous septum morphogenesis	GO:0003149	0.114	1
amino acid salvage	GO:0043102	0.114	1
locomotor rhythm	GO:0045475	0.114	1
T-helper 2 cell cytokine production	GO:0035745	0.114	1
negative regulation of protein localization to cell surface	GO:2000009	0.114	1
protein-chromophore linkage	GO:0018298	0.114	1
positive regulation of protein complex assembly	GO:0031334	0.115	3
cell fate commitment	GO:0045165	0.116	3
regulation of immune system process	GO:0002682	0.116	7
regulation of protein complex assembly	GO:0043254	0.116	4
renal tubule development	GO:0061326	0.116	2
nucleoside diphosphate biosynthetic process	GO:0009133	0.116	1
positive regulation of developmental pigmentation	GO:0048087	0.116	1
positive regulation of centrosome duplication	GO:0010825	0.116	1
negative regulation of cardiac muscle cell apoptotic process	GO:0010667	0.116	1
L-methionine biosynthetic process	GO:0071265	0.116	1
Mo-molybdopterin cofactor biosynthetic process	GO:0006777	0.116	1
Mo-molybdopterin cofactor metabolic process	GO:0019720	0.116	1
mesenchymal to epithelial transition involved in metanephros morphogenesis	GO:0003337	0.116	1
negative regulation of endoplasmic reticulum unfolded protein response	GO:1900102	0.116	1
regulation of cardioblast differentiation	GO:0051890	0.116	1

rRNA base methylation	GO:0070475	0.116	1
positive regulation of macrophage differentiation	GO:0045651	0.116	1
mesodermal cell fate specification	GO:0007501	0.116	1
regulation of glial cell proliferation	GO:0060251	0.116	1
eyelid development in camera-type eye	GO:0061029	0.116	1
negative regulation of cytoplasmic translation	GO:2000766	0.116	1
regulation of mucus secretion	GO:0070255	0.116	1
neural crest formation	GO:0014029	0.116	1
MyD88-independent toll-like receptor signaling pathway	GO:0002756	0.116	1
negative regulation of transcription from RNA polymerase II promoter in response to stress	GO:0097201	0.116	1
plasma membrane to endosome transport	GO:0048227	0.116	1
macrophage activation involved in immune response	GO:0002281	0.116	1
response to organic substance	GO:0010033	0.116	12
embryonic skeletal system morphogenesis	GO:0048704	0.116	2
cellular response to unfolded protein	GO:0034620	0.116	2
regulation of DNA binding	GO:0051101	0.116	2
regulation of leukocyte activation	GO:0002694	0.116	4
establishment of localization in cell	GO:0051649	0.116	12
metabolic process	GO:0008152	0.116	51
kidney development	GO:0001822	0.116	3
positive regulation of biological process	GO:0048518	0.116	22
sensory organ morphogenesis	GO:0090596	0.118	3
regulation of transmembrane transport	GO:0034762	0.118	4
cell adhesion	GO:0007155	0.118	8
toll-like receptor signaling pathway	GO:0002224	0.118	2
regulation of osteoblast differentiation	GO:0045667	0.118	2

fibril organization	GO:0097435	0.118	5
cellular response to prostaglandin E stimulus	GO:0071380	0.118	1
Peyer's patch development	GO:0048541	0.118	1
positive regulation of synapse maturation	GO:0090129	0.118	1
negative regulation of oligodendrocyte differentiation	GO:0048715	0.118	1
molybdopterin cofactor metabolic process	GO:0043545	0.118	1
regulation of pigment cell differentiation	GO:0050932	0.118	1
negative regulation of striated muscle cell apoptotic process	GO:0010664	0.118	1
mucosal-associated lymphoid tissue development	GO:0048537	0.118	1
negative regulation of neuron migration	GO:2001223	0.118	1
prosthetic group metabolic process	GO:0051189	0.118	1
CD4-positive, alpha-beta T cell cytokine production	GO:0035743	0.118	1
regulation of PERK-mediated unfolded protein response	GO:1903897	0.118	1
regulation of proteolysis involved in cellular protein catabolic process	GO:1903050	0.119	3
biological adhesion	GO:0022610	0.12	8
cellular response to lipid	GO:0071396	0.12	4
single-organism transport	GO:0044765	0.121	13
single-organism behavior	GO:0044708	0.122	3
positive regulation of extracellular matrix organization	GO:1903055	0.122	2
positive regulation of endoplasmic reticulum stress-induced intrinsic apoptotic signaling pathway	GO:1902237	0.122	1
response to isoquinoline alkaloid	GO:0014072	0.122	1
response to morphine	GO:0043278	0.122	1
negative regulation of multicellular organism growth	GO:0040015	0.122	1
cardiac cell fate commitment	GO:0060911	0.122	1
loop of Henle development	GO:0072070	0.122	1
distal tubule development	GO:0072017	0.122	1

dosage compensation by inactivation of X chromosome	GO:0009048	0.122	1
cellular response to prostaglandin stimulus	GO:0071379	0.122	1
positive regulation of astrocyte differentiation	GO:0048711	0.122	1
response to unfolded protein	GO:0006986	0.123	2
regulation of cell projection organization	GO:0031344	0.124	4
nephron epithelium development	GO:0072009	0.126	2
morphogenesis of an epithelium	GO:0002009	0.128	4
regulation of cell activation	GO:0050865	0.128	4
locomotory exploration behavior	GO:0035641	0.128	1
cellular response to lithium ion	GO:0071285	0.128	1
metanephric renal vesicle morphogenesis	GO:0072283	0.128	1
response to lithium ion	GO:0010226	0.128	1
negative regulation of transcription regulatory region DNA binding	GO:2000678	0.128	1
renal system development	GO:0072001	0.128	3
multicellular organismal process	GO:0032501	0.132	28
regulation of cell morphogenesis	GO:0022604	0.133	4
cellular response to topologically incorrect protein	GO:0035967	0.133	2
PERK-mediated unfolded protein response	GO:0036499	0.133	1
muscle cell fate commitment	GO:0042693	0.133	1
pyrimidine deoxyribonucleotide biosynthetic process	GO:0009221	0.133	1
regulation of cardiac muscle cell apoptotic process	GO:0010665	0.133	1
regulation of synapse maturation	GO:0090128	0.133	1
response to prostaglandin E	GO:0034695	0.133	1
dosage compensation	GO:0007549	0.133	1
mucus secretion	GO:0070254	0.133	1
regulation of calcium ion transmembrane transport	GO:1903169	0.134	2

cerebral cortex development	GO:0021987	0.134	2
regulation of cellular protein catabolic process	GO:1903362	0.134	3
gene expression	GO:0010467	0.135	24
T cell aggregation	GO:0070489	0.136	3
neural tube formation	GO:0001841	0.136	2
cAMP biosynthetic process	GO:0006171	0.136	2
regulation of developmental growth	GO:0048638	0.136	3
lymphocyte aggregation	GO:0071593	0.136	3
adult heart development	GO:0007512	0.136	1
positive regulation of type 2 immune response	GO:0002830	0.136	1
epithelial cilium movement	GO:0003351	0.136	1
limb bud formation	GO:0060174	0.136	1
negative regulation of myotube differentiation	GO:0010832	0.136	1
positive regulation of collateral sprouting	GO:0048672	0.136	1
response to prostaglandin	GO:0034694	0.136	1
regulation of striated muscle cell apoptotic process	GO:0010662	0.136	1
positive regulation of T cell cytokine production	GO:0002726	0.136	1
ear morphogenesis	GO:0042471	0.138	2
cardiocyte differentiation	GO:0035051	0.138	2
mesenchymal cell development	GO:0014031	0.138	2
B cell activation	GO:0042113	0.138	3
regulation of wound healing	GO:0061041	0.138	2
negative regulation of immune response	GO:0050777	0.138	2
renal vesicle morphogenesis	GO:0072077	0.138	1
2'-deoxyribonucleotide biosynthetic process	GO:0009265	0.138	1
mesenchymal to epithelial transition	GO:0060231	0.138	1

cardiac muscle cell apoptotic process	GO:0010659	0.138	1
positive regulation of centrosome cycle	GO:0046607	0.138	1
regulation of store-operated calcium entry	GO:2001256	0.138	1
deoxyribose phosphate biosynthetic process	GO:0046385	0.138	1
ventricular trabecula myocardium morphogenesis	GO:0003222	0.138	1
kidney mesenchyme development	GO:0072074	0.138	1
nuclear pore organization	GO:0006999	0.138	1
negative regulation of sodium ion transport	GO:0010766	0.138	1
renal vesicle development	GO:0072087	0.138	1
sensory organ development	GO:0007423	0.138	4
cell-substrate adhesion	GO:0031589	0.138	3
leukocyte aggregation	GO:0070486	0.141	3
regulation of anatomical structure size	GO:0090066	0.142	4
muscle cell proliferation	GO:0033002	0.142	2
chondrocyte proliferation	GO:0035988	0.142	1
mammary gland epithelial cell differentiation	GO:0060644	0.142	1
ovulation	GO:0030728	0.142	1
atrial septum morphogenesis	GO:0060413	0.142	1
toll-like receptor 9 signaling pathway	GO:0034162	0.142	1
regulation of DNA damage checkpoint	GO:2000001	0.142	1
nucleoside transmembrane transport	GO:1901642	0.142	1
negative regulation of dendrite development	GO:2000171	0.142	1
striated muscle cell apoptotic process	GO:0010658	0.142	1
cellular response to inorganic substance	GO:0071241	0.144	2
positive regulation of actin filament polymerization	GO:0030838	0.146	2
neuron development	GO:0048666	0.146	6

mesodermal cell fate commitment	GO:0001710	0.146	1
lagging strand elongation	GO:0006273	0.146	1
cardiac myofibril assembly	GO:0055003	0.146	1
negative regulation of muscle cell apoptotic process	GO:0010656	0.146	1
pyrimidine deoxyribonucleotide metabolic process	GO:0009219	0.146	1
left/right pattern formation	GO:0060972	0.146	1
regulation of macrophage differentiation	GO:0045649	0.146	1
regulation of cardiac conduction	GO:1903779	0.146	1
regulation of cellular biosynthetic process	GO:0031326	0.146	18
negative regulation of binding	GO:0051100	0.146	2
positive regulation of metabolic process	GO:0009893	0.147	15
muscle organ development	GO:0007517	0.147	3
response to topologically incorrect protein	GO:0035966	0.147	2
single-organism biosynthetic process	GO:0044711	0.147	6
embryonic skeletal system development	GO:0048706	0.148	2
single-organism localization	GO:1902578	0.149	13
glial cell proliferation	GO:0014009	0.15	1
regulation of developmental pigmentation	GO:0048070	0.15	1
lens morphogenesis in camera-type eye	GO:0002089	0.15	1
nephron development	GO:0072006	0.15	2
protein targeting	GO:0006605	0.151	4
positive regulation of cell cycle	GO:0045787	0.151	3
establishment of protein localization to organelle	GO:0072594	0.152	4
regulation of biosynthetic process	GO:0009889	0.152	18
urogenital system development	GO:0001655	0.152	3
cardiovascular system development	GO:0072358	0.152	5

negative regulation of sequence-specific DNA binding transcription factor activity	GO:0043433	0.152	2
response to alcohol	GO:0097305	0.152	2
epithelial cell differentiation	GO:0030855	0.152	4
middle ear morphogenesis	GO:0042474	0.152	1
metanephric nephron tubule development	GO:0072234	0.152	1
nucleoside transport	GO:0015858	0.152	1
atrial septum development	GO:0003283	0.152	1
venous blood vessel development	GO:0060841	0.152	1
positive regulation of toll-like receptor signaling pathway	GO:0034123	0.152	1
cAMP metabolic process	GO:0046058	0.152	2
single-organism intracellular transport	GO:1902582	0.154	6
cellular response to lipopolysaccharide	GO:0071222	0.155	2
RNA metabolic process	GO:0016070	0.156	20
columnar/cuboidal epithelial cell differentiation	GO:0002065	0.156	2
adenylate cyclase-activating dopamine receptor signaling pathway	GO:0007191	0.157	1
signal transduction involved in cell cycle checkpoint	GO:0072395	0.157	1
embryonic epithelial tube formation	GO:0001838	0.157	2
regulation of transcription from RNA polymerase II promoter	GO:0006357	0.157	10
ubiquitin-dependent protein catabolic process	GO:0006511	0.157	5
forebrain development	GO:0030900	0.157	3
neural precursor cell proliferation	GO:0061351	0.157	2
regulation of cytoskeleton organization	GO:0051493	0.157	4
regulation of cellular metabolic process	GO:0031323	0.157	24
epithelial tube formation	GO:0072175	0.157	2
regulation of adaptive immune response	GO:0002819	0.157	2
nucleic acid metabolic process	GO:0090304	0.157	22

response to endogenous stimulus	GO:0009719	0.157	7
cardioblast differentiation	GO:0010002	0.157	1
regulation of hormone biosynthetic process	GO:0046885	0.157	1
regulation of ubiquitin protein ligase activity	GO:1904666	0.157	1
metanephric tubule development	GO:0072170	0.157	1
positive regulation of transcription initiation from RNA polymerase II promoter	GO:0060261	0.157	1
synapse maturation	GO:0060074	0.157	1
methionine biosynthetic process	GO:0009086	0.157	1
positive regulation of cilium assembly	GO:0045724	0.157	1
mammary gland lobule development	GO:0061377	0.157	1
negative regulation of neural precursor cell proliferation	GO:2000178	0.157	1
regulation of translational initiation by eIF2 alpha phosphorylation	GO:0010998	0.157	1
positive regulation of signal transduction by p53 class mediator	GO:1901798	0.157	1
mammary gland alveolus development	GO:0060749	0.157	1
pattern recognition receptor signaling pathway	GO:0002221	0.157	2
kidney epithelium development	GO:0072073	0.157	2
cellular response to molecule of bacterial origin	GO:0071219	0.158	2
stem cell proliferation	GO:0072089	0.159	2
DNA-templated transcription, initiation	GO:0006352	0.159	2
positive regulation of transcription, DNA-templated	GO:0045893	0.16	8
positive regulation of macromolecule metabolic process	GO:0010604	0.16	13
modification-dependent protein catabolic process	GO:0019941	0.16	5
regulation of collateral sprouting	GO:0048670	0.16	1
sympathetic nervous system development	GO:0048485	0.16	1
kinetochore assembly	GO:0051382	0.16	1
cardiac right ventricle morphogenesis	GO:0003215	0.16	1

innate immune response-activating signal transduction	GO:0002758	0.16	2
positive regulation of gene expression	GO:0010628	0.16	9
muscle structure development	GO:0061061	0.161	4
regulation of B cell activation	GO:0050864	0.161	2
regulation of cell-substrate adhesion	GO:0010810	0.162	2
B cell differentiation	GO:0030183	0.162	2
regulation of metabolic process	GO:0019222	0.162	26
pallium development	GO:0021543	0.162	2
cellular glucuronidation	GO:0052695	0.162	1
metanephric nephron morphogenesis	GO:0072273	0.162	1
negative regulation of glial cell differentiation	GO:0045686	0.162	1
outflow tract septum morphogenesis	GO:0003148	0.162	1
phospholipase C-activating dopamine receptor signaling pathway	GO:0060158	0.162	1
regulation of endoplasmic reticulum unfolded protein response	GO:1900101	0.162	1
response to ischemia	GO:0002931	0.162	1
deoxyribonucleotide biosynthetic process	GO:0009263	0.162	1
deoxyribonucleoside triphosphate metabolic process	GO:0009200	0.162	1
wound healing	GO:0042060	0.162	3
peptidyl-serine phosphorylation	GO:0018105	0.162	3
modification-dependent macromolecule catabolic process	GO:0043632	0.162	5
regulation of neuron projection development	GO:0010975	0.164	3
negative regulation of cellular protein metabolic process	GO:0032269	0.164	6
positive regulation of protein polymerization	GO:0032273	0.164	2
metanephric nephron epithelium development	GO:0072243	0.164	1
store-operated calcium entry	GO:0002115	0.164	1
positive regulation of cardiocyte differentiation	GO:1905209	0.164	1

copper ion homeostasis	GO:0055070	0.164	1
negative regulation of peptidyl-serine phosphorylation	GO:0033137	0.164	1
smooth muscle tissue development	GO:0048745	0.164	1
positive regulation of calcium ion transmembrane transporter activity	GO:1901021	0.164	1
embryonic camera-type eye morphogenesis	GO:0048596	0.164	1
regulation of actin cytoskeleton organization	GO:0032956	0.164	3
regulation of cell cycle	GO:0051726	0.165	6
regulation of sequence-specific DNA binding transcription factor activity	GO:0051090	0.165	3
regulation of gene expression	GO:0010468	0.166	18
positive regulation of nucleobase-containing compound metabolic process	GO:0045935	0.166	9
positive regulation of cellular metabolic process	GO:0031325	0.166	13
striated muscle cell development	GO:0055002	0.166	2
regulation of T cell cytokine production	GO:0002724	0.166	1
regulation of cytoplasmic translation	GO:2000765	0.166	1
regulation of translational initiation in response to stress	GO:0043558	0.166	1
photoperiodism	GO:0009648	0.166	1
nucleus localization	GO:0051647	0.166	1
thyroid gland development	GO:0030878	0.166	1
glucuronate metabolic process	GO:0019585	0.166	1
uronic acid metabolic process	GO:0006063	0.166	1
cellular response to estradiol stimulus	GO:0071392	0.166	1
phototransduction	GO:0007602	0.166	1
entrainment of circadian clock by photoperiod	GO:0043153	0.166	1
response to oxygen-containing compound	GO:1901700	0.166	6
positive regulation of nucleic acid-templated transcription	GO:1903508	0.167	8
heterocycle biosynthetic process	GO:0018130	0.167	18

positive regulation of RNA biosynthetic process	GO:1902680	0.167	8
tube formation	GO:0035148	0.167	2
cyclic purine nucleotide metabolic process	GO:0052652	0.167	2
apoptotic process involved in morphogenesis	GO:0060561	0.167	1
innervation	GO:0060384	0.167	1
pyrimidine nucleoside triphosphate biosynthetic process	GO:0009148	0.167	1
regulation of synaptic transmission, GABAergic	GO:0032228	0.167	1
positive regulation of blood pressure	GO:0045777	0.167	1
negative regulation of protein ubiquitination involved in ubiquitin-dependent protein catabolic process	GO:2000059	0.167	1
regulation of fibroblast migration	GO:0010762	0.167	1
kinetochore organization	GO:0051383	0.167	1
regulation of type 2 immune response	GO:0002828	0.167	1
regulation of translation in response to stress	GO:0043555	0.167	1
mammary gland epithelial cell proliferation	GO:0033598	0.167	1
regulation of intrinsic apoptotic signaling pathway by p53 class mediator	GO:1902253	0.167	1
regulation of intrinsic apoptotic signaling pathway	GO:2001242	0.167	2
response to radiation	GO:0009314	0.167	3
transcription from RNA polymerase II promoter	GO:0006366	0.167	10
regulation of epithelial cell migration	GO:0010632	0.167	2
regulation of proteasomal ubiquitin-dependent protein catabolic process	GO:0032434	0.167	2
lymphocyte differentiation	GO:0030098	0.168	3
regulation of axonogenesis	GO:0050770	0.168	2
negative regulation of neuron differentiation	GO:0045665	0.168	2
transforming growth factor beta receptor signaling pathway	GO:0007179	0.168	2
single-organism membrane fusion	GO:0044801	0.168	2
cellular localization	GO:0051641	0.168	13

negative regulation of striated muscle cell differentiation	GO:0051154	0.168	1
regulation of astrocyte differentiation	GO:0048710	0.168	1
cellular response to fatty acid	GO:0071398	0.168	1
positive regulation of heart contraction	GO:0045823	0.168	1
cellular response to biotic stimulus	GO:0071216	0.169	2
RNA processing	GO:0006396	0.17	6
positive regulation of cellular biosynthetic process	GO:0031328	0.17	9
peptidyl-serine modification	GO:0018209	0.17	3
transmembrane receptor protein serine/threonine kinase signaling pathway	GO:0007178	0.17	3
translational initiation	GO:0006413	0.17	2
regulation of translation	GO:0006417	0.17	3
positive regulation of glial cell differentiation	GO:0045687	0.17	1
DNA strand elongation involved in DNA replication	GO:0006271	0.17	1
regulation of oligodendrocyte differentiation	GO:0048713	0.17	1
regulation of branching involved in ureteric bud morphogenesis	GO:0090189	0.17	1
deoxyribose phosphate metabolic process	GO:0019692	0.17	1
exploration behavior	GO:0035640	0.17	1
pharyngeal system development	GO:0060037	0.17	1
2'-deoxyribonucleotide metabolic process	GO:0009394	0.17	1
metanephric epithelium development	GO:0072207	0.17	1
morphogenesis of embryonic epithelium	GO:0016331	0.171	2
cyclic nucleotide biosynthetic process	GO:0009190	0.173	2
regulation of primary metabolic process	GO:0080090	0.173	23
very long-chain fatty acid biosynthetic process	GO:0042761	0.173	1
replication fork processing	GO:0031297	0.173	1
regulation of hormone metabolic process	GO:0032350	0.173	1

circadian behavior	GO:0048512	0.173	1
aorta morphogenesis	GO:0035909	0.173	1
activation of innate immune response	GO:0002218	0.174	2
organelle membrane fusion	GO:0090174	0.175	2
regulation of organelle assembly	GO:1902115	0.175	2
positive regulation of biosynthetic process	GO:0009891	0.175	9
positive regulation of neuron projection development	GO:0010976	0.175	2
negative regulation of protein metabolic process	GO:0051248	0.175	6
methionine metabolic process	GO:0006555	0.175	1
sulfur amino acid biosynthetic process	GO:0000097	0.175	1
regulation of transcription initiation from RNA polymerase II promoter	GO:0060260	0.175	1
programmed cell death involved in cell development	GO:0010623	0.175	1
regulation of protein localization to cell surface	GO:2000008	0.175	1
T cell cytokine production	GO:0002369	0.175	1
regulation of mesonephros development	GO:0061217	0.175	1
negative regulation of ligase activity	GO:0051352	0.175	1
DNA-dependent DNA replication	GO:0006261	0.175	2
muscle cell development	GO:0055001	0.175	2
circadian rhythm	GO:0007623	0.176	2
cell death	GO:0008219	0.176	8
neural tube development	GO:0021915	0.176	2
negative regulation of protein phosphorylation	GO:0001933	0.176	3
blood circulation	GO:0008015	0.176	3
rRNA methylation	GO:0031167	0.176	1
positive regulation of sodium ion transport	GO:0010765	0.176	1
cell proliferation in forebrain	GO:0021846	0.176	1

positive regulation of erythrocyte differentiation	GO:0045648	0.176	1
cell fate commitment involved in formation of primary germ layer	GO:0060795	0.176	1
positive regulation of cation channel activity	GO:2001259	0.176	1
rhythmic behavior	GO:0007622	0.176	1
positive regulation of DNA-templated transcription, initiation	GO:2000144	0.176	1
organic cyclic compound biosynthetic process	GO:1901362	0.178	18
positive regulation of RNA metabolic process	GO:0051254	0.178	8
protein complex assembly	GO:0006461	0.178	5
protein complex biogenesis	GO:0070271	0.179	5
response to lipid	GO:0033993	0.179	4
regulation of actin filament-based process	GO:0032970	0.179	3
regulation of B cell differentiation	GO:0045577	0.179	1
regulation of muscle cell apoptotic process	GO:0010660	0.179	1
response to fatty acid	GO:0070542	0.179	1
natural killer cell differentiation	GO:0001779	0.179	1
proteolysis involved in cellular protein catabolic process	GO:0051603	0.179	5
circulatory system process	GO:0003013	0.179	3
glial cell differentiation	GO:0010001	0.181	2
entrainment of circadian clock	GO:0009649	0.181	1
positive regulation of adenylate cyclase activity involved in G-protein coupled receptor signaling pathway	GO:0010579	0.181	1
collateral sprouting	GO:0048668	0.181	1
pyrimidine nucleoside triphosphate metabolic process	GO:0009147	0.181	1
type 2 immune response	GO:0042092	0.181	1
regulation of adenylate cyclase activity involved in G-protein coupled receptor signaling pathway	GO:0010578	0.181	1
toll-like receptor 4 signaling pathway	GO:0034142	0.181	1
regulation of cellular component organization	GO:0051128	0.182	10

homotypic cell-cell adhesion	GO:0034109	0.183	3
circulatory system development	GO:0072359	0.183	5
regulation of macromolecule metabolic process	GO:0060255	0.183	23
regulation of anatomical structure morphogenesis	GO:0022603	0.183	5
positive regulation of apoptotic signaling pathway	GO:2001235	0.183	2
regulation of biological quality	GO:0065008	0.183	14
synaptic transmission, GABAergic	GO:0051932	0.183	1
embryonic eye morphogenesis	GO:0048048	0.183	1
regulation of endoplasmic reticulum stress-induced intrinsic apoptotic signaling pathway	GO:1902235	0.183	1
positive regulation of cell cycle arrest	GO:0071158	0.183	1
cellular response to estrogen stimulus	GO:0071391	0.183	1
metanephros morphogenesis	GO:0003338	0.183	1
signal release	GO:0023061	0.184	3
BMP signaling pathway	GO:0030509	0.184	2
positive regulation of fibril organization	GO:1902905	0.184	2
cellular response to endogenous stimulus	GO:0071495	0.184	6
GTP metabolic process	GO:0046039	0.184	1
fibroblast migration	GO:0010761	0.184	1
cardiac atrium morphogenesis	GO:0003209	0.184	1
detection of visible light	GO:0009584	0.184	1
olfactory bulb development	GO:0021772	0.184	1
positive regulation of Rho protein signal transduction	GO:0035025	0.184	1
photoreceptor cell maintenance	GO:0045494	0.184	1
DNA strand elongation	GO:0022616	0.184	1
cofactor transport	GO:0051181	0.184	1
mesodermal cell differentiation	GO:0048333	0.184	1

regulation of cell cycle checkpoint	GO:1901976	0.184	1
regulation of localization	GO:0032879	0.185	10
regulation of lymphocyte proliferation	GO:0050670	0.185	2
regulation of cellular component size	GO:0032535	0.185	3
regulation of cellular macromolecule biosynthetic process	GO:2000112	0.185	16
regulation of hormone levels	GO:0010817	0.185	3
regulation of mononuclear cell proliferation	GO:0032944	0.185	2
nucleocytoplasmic transport	GO:0006913	0.185	3
cellular macromolecule catabolic process	GO:0044265	0.185	6
negative regulation of ubiquitin-protein transferase activity	GO:0051444	0.185	1
centromere complex assembly	GO:0034508	0.185	1
positive regulation of lipid catabolic process	GO:0050996	0.185	1
regulation of cardiocyte differentiation	GO:1905207	0.185	1
regulation of cardiac muscle cell proliferation	GO:0060043	0.185	1
peptide cross-linking	GO:0018149	0.185	1
apoptotic process involved in development	GO:1902742	0.185	1
muscle cell apoptotic process	GO:0010657	0.185	1
cell-matrix adhesion	GO:0007160	0.185	2
regulation of cellular amide metabolic process	GO:0034248	0.185	3
regulation of extracellular matrix organization	GO:1903053	0.186	2
cellular response to retinoic acid	GO:0071300	0.187	1
macrophage differentiation	GO:0030225	0.187	1
DNA-dependent DNA replication maintenance of fidelity	GO:0045005	0.187	1
embryonic camera-type eye development	GO:0031076	0.187	1
olfactory lobe development	GO:0021988	0.187	1
melanocyte differentiation	GO:0030318	0.187	1

RNA polymerase II transcriptional preinitiation complex assembly	GO:0051123	0.187	1
adipose tissue development	GO:0060612	0.187	1
positive regulation of smooth muscle cell proliferation	GO:0048661	0.187	1
positive regulation of intracellular signal transduction	GO:1902533	0.187	5
cellular response to transforming growth factor beta stimulus	GO:0071560	0.187	2
response to BMP	GO:0071772	0.187	2
cellular response to BMP stimulus	GO:0071773	0.187	2
regulation of hormone secretion	GO:0046883	0.188	2
negative regulation of phosphorylation	GO:0042326	0.188	3
response to transforming growth factor beta	GO:0071559	0.189	2
regulation of leukocyte proliferation	GO:0070663	0.189	2
oligodendrocyte development	GO:0014003	0.189	1
cardiac atrium development	GO:0003230	0.189	1
cellular protein catabolic process	GO:0044257	0.19	5
negative regulation of cell migration	GO:0030336	0.192	2
cyclic nucleotide metabolic process	GO:0009187	0.192	2
positive regulation of receptor activity	GO:2000273	0.192	1
regulation of protein catabolic process	GO:0042176	0.194	3
regulation of immune response	GO:0050776	0.195	4
locomotory behavior	GO:0007626	0.195	2
organic substance catabolic process	GO:1901575	0.195	9
aspartate family amino acid biosynthetic process	GO:0009067	0.195	1
positive regulation of gliogenesis	GO:0014015	0.195	1
hippo signaling	GO:0035329	0.195	1
nucleobase-containing compound biosynthetic process	GO:0034654	0.196	17
regulation of microtubule cytoskeleton organization	GO:0070507	0.198	2

deoxyribonucleotide metabolic process	GO:0009262	0.199	1
negative regulation of growth	GO:0045926	0.199	2
cellular protein complex assembly	GO:0043623	0.2	3
leukocyte cell-cell adhesion	GO:0007159	0.2	3
movement of cell or subcellular component	GO:0006928	0.201	8
regulation of voltage-gated calcium channel activity	GO:1901385	0.201	1
regulation of cardiac muscle tissue growth	GO:0055021	0.201	1
metanephric nephron development	GO:0072210	0.201	1
cytosolic transport	GO:0016482	0.202	4
regulation of actin filament polymerization	GO:0030833	0.203	2
cell cycle process	GO:0022402	0.204	6
positive regulation of transcription from RNA polymerase II promoter	GO:0045944	0.204	6
response to estradiol	GO:0032355	0.204	1
regulation of DNA-templated transcription, initiation	GO:2000142	0.204	1
regulation of neuron migration	GO:2001222	0.204	1
positive regulation of cytokine production involved in immune response	GO:0002720	0.204	1
response to ethanol	GO:0045471	0.204	1
ear development	GO:0043583	0.204	2
organonitrogen compound catabolic process	GO:1901565	0.204	6
organelle fusion	GO:0048284	0.205	2
negative regulation of cell motility	GO:2000146	0.206	2
cell projection organization	GO:0030030	0.206	6
regulation of transcription regulatory region DNA binding	GO:2000677	0.206	1
positive regulation of T cell mediated immunity	GO:0002711	0.206	1
negative regulation of adaptive immune response	GO:0002820	0.206	1
positive regulation of cytokinesis	GO:0032467	0.206	1

regulation of macromolecule biosynthetic process	GO:0010556	0.211	16
single-organism organelle organization	GO:1902589	0.213	8
very long-chain fatty acid metabolic process	GO:0000038	0.213	1
vasodilation	GO:0042311	0.213	1
detection of light stimulus	GO:0009583	0.213	1
positive regulation of macromolecule biosynthetic process	GO:0010557	0.214	8
aromatic compound biosynthetic process	GO:0019438	0.214	17
autonomic nervous system development	GO:0048483	0.216	1
regulation of heart growth	GO:0060420	0.216	1
protein-DNA complex assembly	GO:0065004	0.216	2
positive regulation of innate immune response	GO:0045089	0.216	2
posttranscriptional regulation of gene expression	GO:0010608	0.217	3
peptidyl-tyrosine phosphorylation	GO:0018108	0.217	3
ER-nucleus signaling pathway	GO:0006984	0.217	1
epithelial cell differentiation involved in kidney development	GO:0035850	0.217	1
sulfur compound transport	GO:0072348	0.217	1
negative regulation of osteoblast differentiation	GO:0045668	0.217	1
negative regulation of response to endoplasmic reticulum stress	GO:1903573	0.217	1
spleen development	GO:0048536	0.217	1
positive regulation of calcium ion transmembrane transport	GO:1904427	0.217	1
regulation of actin filament length	GO:0030832	0.219	2
regulation of actin polymerization or depolymerization	GO:0008064	0.219	2
gliogenesis	GO:0042063	0.219	2
negative regulation of STAT cascade	GO:1904893	0.219	1
rRNA modification	GO:0000154	0.219	1
maturation of 5.8S rRNA	GO:0000460	0.219	1

regulation of focal adhesion assembly	GO:0051893	0.219	1
positive regulation of stem cell differentiation	GO:2000738	0.219	1
regulation of cell-substrate junction assembly	GO:0090109	0.219	1
peptidyl-tyrosine modification	GO:0018212	0.221	3
actin filament polymerization	GO:0030041	0.221	2
heterophilic cell-cell adhesion via plasma membrane cell adhesion molecules	GO:0007157	0.221	1
positive regulation of cellular carbohydrate metabolic process	GO:0010676	0.221	1
positive regulation of insulin secretion	GO:0032024	0.221	1
regulation of sodium ion transmembrane transport	GO:1902305	0.221	1
positive regulation of myeloid leukocyte differentiation	GO:0002763	0.221	1
epithelial cell migration	GO:0010631	0.222	2
regulation of microtubule-based process	GO:0032886	0.223	2
regulation of peptidyl-tyrosine phosphorylation	GO:0050730	0.223	2
enzyme linked receptor protein signaling pathway	GO:0007167	0.223	5
epithelium migration	GO:0090132	0.223	2
positive regulation of adenylate cyclase activity	GO:0045762	0.223	1
pyrimidine nucleotide biosynthetic process	GO:0006221	0.223	1
pigment cell differentiation	GO:0050931	0.223	1
positive regulation of BMP signaling pathway	GO:0030513	0.223	1
toxin transport	GO:1901998	0.223	1
organophosphate biosynthetic process	GO:0090407	0.224	4
T cell activation	GO:0042110	0.224	3
DNA-templated transcriptional preinitiation complex assembly	GO:0070897	0.225	1
negative regulation of JAK-STAT cascade	GO:0046426	0.225	1
cardiac muscle cell proliferation	GO:0060038	0.225	1
regulation of erythrocyte differentiation	GO:0045646	0.225	1

heart looping	GO:0001947	0.225	1
T cell differentiation	GO:0030217	0.225	2
negative regulation of cellular component movement	GO:0051271	0.225	2
regulation of molecular function	GO:0065009	0.225	12
cell projection morphogenesis	GO:0048858	0.225	4
response to lipopolysaccharide	GO:0032496	0.225	2
cell cycle checkpoint	GO:0000075	0.225	2
cellular metabolic compound salvage	GO:0043094	0.225	1
negative regulation of axonogenesis	GO:0050771	0.225	1
lamellipodium assembly	GO:0030032	0.225	1
regulation of cilium assembly	GO:1902017	0.225	1
somatic stem cell population maintenance	GO:0035019	0.225	1
metaphase plate congression	GO:0051310	0.225	1
nuclear envelope organization	GO:0006998	0.225	1
tissue migration	GO:0090130	0.227	2
aorta development	GO:0035904	0.228	1
positive regulation of wound healing	GO:0090303	0.228	1
regulation of glial cell differentiation	GO:0045685	0.228	1
calcium ion transport	GO:0006816	0.229	3
negative regulation of hemostasis	GO:1900047	0.23	1
regulation of kidney development	GO:0090183	0.23	1
negative regulation of blood coagulation	GO:0030195	0.23	1
determination of heart left/right asymmetry	GO:0061371	0.23	1
epithelial cell morphogenesis	GO:0003382	0.23	1
retina homeostasis	GO:0001895	0.23	1
locomotion	GO:0040011	0.23	7

regulation of transcription, DNA-templated	GO:0006355	0.231	14
negative regulation of cell cycle	GO:0045786	0.231	3
macromolecule modification	GO:0043412	0.231	18
regulation of transport	GO:0051049	0.231	7
sarcomere organization	GO:0045214	0.231	1
cellular response to ketone	GO:1901655	0.231	1
regulation of toll-like receptor signaling pathway	GO:0034121	0.231	1
anterior/posterior axis specification	GO:0009948	0.231	1
protein localization to cell surface	GO:0034394	0.231	1
regulation of centrosome duplication	GO:0010824	0.231	1
negative regulation of lymphocyte differentiation	GO:0045620	0.231	1
regulation of insulin secretion involved in cellular response to glucose stimulus	GO:0061178	0.231	1
cellular senescence	GO:0090398	0.231	1
negative regulation of locomotion	GO:0040013	0.232	2
axon guidance	GO:0007411	0.232	2
regulation of proteasomal protein catabolic process	GO:0061136	0.232	2
peptide transport	GO:0015833	0.232	7
regulation of adherens junction organization	GO:1903391	0.232	1
ventricular cardiac muscle tissue morphogenesis	GO:0055010	0.232	1
positive regulation of cell projection organization	GO:0031346	0.232	2
response to light stimulus	GO:0009416	0.232	2
cellular component assembly involved in morphogenesis	GO:0010927	0.232	2
cell part morphogenesis	GO:0032990	0.234	4
neuron projection guidance	GO:0097485	0.234	2
skeletal system morphogenesis	GO:0048705	0.234	2
negative regulation of coagulation	GO:0050819	0.234	1

positive regulation of osteoblast differentiation	GO:0045669	0.234	1
morphogenesis of an epithelial sheet	GO:0002011	0.234	1
response to molecule of bacterial origin	GO:0002237	0.235	2
membrane fusion	GO:0061025	0.235	2
negative regulation of response to stimulus	GO:0048585	0.235	6
rhythmic process	GO:0048511	0.235	2
regulation of protein polymerization	GO:0032271	0.235	2
regulation of myotube differentiation	GO:0010830	0.235	1
lipopolysaccharide-mediated signaling pathway	GO:0031663	0.235	1
sulfur amino acid metabolic process	GO:0000096	0.235	1
positive regulation of Ras protein signal transduction	GO:0046579	0.235	1
pyrimidine-containing compound biosynthetic process	GO:0072528	0.235	1
central nervous system development	GO:0007417	0.236	4
connective tissue development	GO:0061448	0.236	2
nucleotide biosynthetic process	GO:0009165	0.236	3
hormone secretion	GO:0046879	0.236	2
cellular process	GO:0009987	0.236	64
collagen fibril organization	GO:0030199	0.236	1
pyrimidine nucleotide metabolic process	GO:0006220	0.236	1
negative regulation of peptidyl-tyrosine phosphorylation	GO:0050732	0.236	1
negative regulation of epithelial cell migration	GO:0010633	0.236	1
positive regulation of ion transmembrane transporter activity	GO:0032414	0.236	1
regulation of lipid catabolic process	GO:0050994	0.236	1
mesenchymal cell proliferation	GO:0010463	0.236	1
regulation of chondrocyte differentiation	GO:0032330	0.236	1
positive regulation of fat cell differentiation	GO:0045600	0.236	1

immune system process	GO:0002376	0.237	9
regulation of innate immune response	GO:0045088	0.238	2
guanosine-containing compound metabolic process	GO:1901068	0.238	1
cardiac muscle tissue growth	GO:0055017	0.238	1
endosome to lysosome transport	GO:0008333	0.238	1
nucleoside phosphate biosynthetic process	GO:1901293	0.238	3
sodium ion transport	GO:0006814	0.238	2
amide transport	GO:0042886	0.238	7
regulation of cell proliferation	GO:0042127	0.239	6
positive regulation of blood circulation	GO:1903524	0.239	1
cilium movement	GO:0003341	0.239	1
secretion by tissue	GO:0032941	0.239	1
artery morphogenesis	GO:0048844	0.239	1
protein-DNA complex subunit organization	GO:0071824	0.24	2
regulation of nucleic acid-templated transcription	GO:1903506	0.241	14
cell-substrate adherens junction assembly	GO:0007045	0.241	1
positive regulation of epithelial cell differentiation	GO:0030858	0.241	1
positive regulation of carbohydrate metabolic process	GO:0045913	0.241	1
focal adhesion assembly	GO:0048041	0.241	1
regulation of RNA biosynthetic process	GO:2001141	0.242	14
embryonic heart tube morphogenesis	GO:0003143	0.244	1
dopamine receptor signaling pathway	GO:0007212	0.244	1
insulin secretion involved in cellular response to glucose stimulus	GO:0035773	0.244	1
macrophage activation	GO:0042116	0.244	1
hormone transport	GO:0009914	0.244	2
response to abiotic stimulus	GO:0009628	0.245	4

positive regulation of small GTPase mediated signal transduction	GO:0051057	0.245	1
developmental pigmentation	GO:0048066	0.245	1
regulation of morphogenesis of a branching structure	GO:0060688	0.245	1
negative regulation of muscle cell differentiation	GO:0051148	0.245	1
regulation of transcription from RNA polymerase II promoter in response to stress	GO:0043618	0.245	1
regulation of signal transduction by p53 class mediator	GO:1901796	0.245	1
embryonic cranial skeleton morphogenesis	GO:0048701	0.245	1
hemopoiesis	GO:0030097	0.245	4
response to endoplasmic reticulum stress	GO:0034976	0.245	2
single organismal cell-cell adhesion	GO:0016337	0.246	3
metal ion transport	GO:0030001	0.246	5
striated muscle cell proliferation	GO:0014855	0.246	1
negative regulation of wound healing	GO:0061045	0.246	1
regulation of cardiac muscle tissue development	GO:0055024	0.246	1
hormone biosynthetic process	GO:0042446	0.246	1
cellular protein modification process	GO:0006464	0.246	17
protein modification process	GO:0036211	0.246	17
protein catabolic process	GO:0030163	0.246	5
cell proliferation	GO:0008283	0.247	7
leukocyte differentiation	GO:0002521	0.247	3
negative regulation of lymphocyte proliferation	GO:0050672	0.247	1
negative regulation of mononuclear cell proliferation	GO:0032945	0.247	1
heart growth	GO:0060419	0.247	1
positive regulation of peptide hormone secretion	GO:0090277	0.247	1
neuromuscular junction development	GO:0007528	0.247	1
positive regulation of signal transduction	GO:0009967	0.248	6

macromolecule catabolic process	GO:0009057	0.249	6
regulation of cell cycle arrest	GO:0071156	0.249	1
branching involved in ureteric bud morphogenesis	GO:0001658	0.249	1
regulation of ligase activity	GO:0051340	0.249	1
regulation of T cell mediated immunity	GO:0002709	0.249	1
intracellular transport	GO:0046907	0.25	8
regulation of growth	GO:0040008	0.25	3
negative regulation of leukocyte proliferation	GO:0070664	0.251	1
cardiac conduction	GO:0061337	0.251	1
CD4-positive, alpha-beta T cell differentiation	GO:0043367	0.251	1
positive regulation of stem cell proliferation	GO:2000648	0.251	1
positive regulation of lyase activity	GO:0051349	0.251	1
striated muscle cell differentiation	GO:0051146	0.251	2
negative regulation of molecular function	GO:0044092	0.251	5
response to external stimulus	GO:0009605	0.251	8
nitrogen compound transport	GO:0071705	0.252	8
regulation of cellular protein metabolic process	GO:0032268	0.252	10
developmental maturation	GO:0021700	0.252	2
lens development in camera-type eye	GO:0002088	0.252	1
positive regulation of transporter activity	GO:0032411	0.252	1
positive regulation of cyclase activity	GO:0031281	0.252	1
actin polymerization or depolymerization	GO:0008154	0.253	2
regulation of cardiac muscle contraction	GO:0055117	0.253	1
regulation of centrosome cycle	GO:0046605	0.253	1
regulation of release of sequestered calcium ion into cytosol	GO:0051279	0.253	1
Golgi to plasma membrane transport	GO:0006893	0.253	1

cytoplasmic microtubule organization	GO:0031122	0.253	1
transcription, DNA-templated	GO:0006351	0.255	14
regulation of homeostatic process	GO:0032844	0.255	2
intrinsic apoptotic signaling pathway in response to endoplasmic reticulum stress	GO:0070059	0.255	1
response to estrogen	GO:0043627	0.255	1
aspartate family amino acid metabolic process	GO:0009066	0.255	1
ovulation cycle process	GO:0022602	0.255	1
regulation of DNA-templated transcription in response to stress	GO:0043620	0.255	1
response to retinoic acid	GO:0032526	0.255	1
adherens junction assembly	GO:0034333	0.257	1
regulation of ubiquitin-protein transferase activity	GO:0051438	0.257	1
endoplasmic reticulum organization	GO:0007029	0.257	1
protein monoubiquitination	GO:0006513	0.257	1
negative regulation of cell death	GO:0060548	0.257	4
divalent metal ion transport	GO:0070838	0.257	3
cranial skeletal system development	GO:1904888	0.259	1
regulation of calcium ion import	GO:0090279	0.259	1
regulation of smooth muscle cell proliferation	GO:0048660	0.259	1
positive regulation of nitrogen compound metabolic process	GO:0051173	0.259	9
divalent inorganic cation transport	GO:0072511	0.259	3
negative regulation of phosphorus metabolic process	GO:0010563	0.26	3
negative regulation of phosphate metabolic process	GO:0045936	0.26	3
cardiac muscle tissue morphogenesis	GO:0055008	0.26	1
positive regulation of synapse assembly	GO:0051965	0.26	1
regulation of cell junction assembly	GO:1901888	0.26	1
positive regulation of immune system process	GO:0002684	0.26	4

regulation of cell death	GO:0010941	0.26	6
positive regulation of secretion by cell	GO:1903532	0.26	2
endosomal transport	GO:0016197	0.26	2
regulation of catabolic process	GO:0009894	0.26	4
actin cytoskeleton organization	GO:0030036	0.26	4
CD4-positive, alpha-beta T cell activation	GO:0035710	0.26	1
cell-substrate junction assembly	GO:0007044	0.26	1
mesonephric tubule morphogenesis	GO:0072171	0.26	1
ureteric bud morphogenesis	GO:0060675	0.26	1
lamellipodium organization	GO:0097581	0.26	1
negative regulation of cation transmembrane transport	GO:1904063	0.26	1
embryonic heart tube development	GO:0035050	0.26	1
protein transport	GO:0015031	0.26	7
astrocyte differentiation	GO:0048708	0.262	1
myeloid cell activation involved in immune response	GO:0002275	0.262	1
calcium ion import into cytosol	GO:1902656	0.262	1
single organism cell adhesion	GO:0098602	0.262	3
regulation of cell cycle phase transition	GO:1901987	0.262	2
synapse organization	GO:0050808	0.263	2
protein localization to organelle	GO:0033365	0.263	4
neuron fate commitment	GO:0048663	0.263	1
ovulation cycle	GO:0042698	0.263	1
regulation of cartilage development	GO:0061035	0.263	1
lung morphogenesis	GO:0060425	0.263	1
camera-type eye development	GO:0043010	0.264	2
regulation of lipid metabolic process	GO:0019216	0.264	2

hematopoietic or lymphoid organ development	GO:0048534	0.265	4
regulation of cytokine production involved in immune response	GO:0002718	0.265	1
smooth muscle cell proliferation	GO:0048659	0.265	1
regulation of protein localization	GO:0032880	0.266	4
nucleic acid-templated transcription	GO:0097659	0.267	14
establishment of chromosome localization	GO:0051303	0.267	1
positive regulation of organelle assembly	GO:1902117	0.267	1
myofibril assembly	GO:0030239	0.267	1
skeletal muscle cell differentiation	GO:0035914	0.267	1
nerve development	GO:0021675	0.267	1
macromolecular complex subunit organization	GO:0043933	0.267	9
positive regulation of organelle organization	GO:0010638	0.267	3
response to ketone	GO:1901654	0.269	1
chromosome localization	GO:0050000	0.269	1
RNA biosynthetic process	GO:0032774	0.272	14
regulation of cell morphogenesis involved in differentiation	GO:0010769	0.272	2
lymphocyte proliferation	GO:0046651	0.272	2
mononuclear cell proliferation	GO:0032943	0.273	2
intrinsic apoptotic signaling pathway	GO:0097193	0.273	2
regulation of calcium ion transport into cytosol	GO:0010522	0.273	1
body fluid secretion	GO:0007589	0.273	1
nucleotide metabolic process	GO:0009117	0.273	4
oligodendrocyte differentiation	GO:0048709	0.275	1
mammary gland epithelium development	GO:0061180	0.275	1
regulation of calcium ion transmembrane transporter activity	GO:1901019	0.275	1
negative regulation of transferase activity	GO:0051348	0.275	2

brain development	GO:0007420	0.275	3
positive regulation of defense response	GO:0031349	0.275	2
positive regulation of secretion	GO:0051047	0.275	2
mesoderm formation	GO:0001707	0.275	1
positive regulation of axonogenesis	GO:0050772	0.275	1
positive regulation of cation transmembrane transport	GO:1904064	0.275	1
negative regulation of NF-kappaB transcription factor activity	GO:0032088	0.275	1
regulation of adenylate cyclase activity	GO:0045761	0.275	1
positive regulation of cAMP biosynthetic process	GO:0030819	0.275	1
response to ammonium ion	GO:0060359	0.275	1
nephron tubule morphogenesis	GO:0072078	0.275	1
regulation of organ growth	GO:0046620	0.277	1
nephron epithelium morphogenesis	GO:0072088	0.277	1
response to inorganic substance	GO:0010035	0.277	2
nucleoside phosphate metabolic process	GO:0006753	0.277	4
response to wounding	GO:0009611	0.277	3
regulation of nucleobase-containing compound metabolic process	GO:0019219	0.278	15
regulation of RNA metabolic process	GO:0051252	0.278	14
lipid storage	GO:0019915	0.278	1
negative regulation of ion transmembrane transport	GO:0034766	0.278	1
nephron morphogenesis	GO:0072028	0.278	1
regulation of protein ubiquitination involved in ubiquitin-dependent protein catabolic process	GO:2000058	0.278	1
regulation of response to stress	GO:0080134	0.28	5
vasculogenesis	GO:0001570	0.28	1
pyrimidine-containing compound metabolic process	GO:0072527	0.28	1
regulation of body fluid levels	GO:0050878	0.281	2

negative regulation of ossification	GO:0030279	0.282	1
response to alkaloid	GO:0043279	0.282	1
metal ion homeostasis	GO:0055065	0.282	3
cellular response to growth factor stimulus	GO:0071363	0.282	3
regulation of striated muscle contraction	GO:0006942	0.282	1
cell fate specification	GO:0001708	0.282	1
mesoderm morphogenesis	GO:0048332	0.282	1
renal tubule morphogenesis	GO:0061333	0.282	1
regulation of neural precursor cell proliferation	GO:2000177	0.282	1
negative regulation of proteolysis involved in cellular protein catabolic process	GO:1903051	0.282	1
regulation of binding	GO:0051098	0.282	2
leukocyte proliferation	GO:0070661	0.282	2
ameboidal-type cell migration	GO:0001667	0.283	2
regulation of cell adhesion	GO:0030155	0.284	3
cellular response to light stimulus	GO:0071482	0.284	1
protein localization	GO:0008104	0.285	9
regulation of protein modification process	GO:0031399	0.286	7
developmental growth	GO:0048589	0.286	3
positive regulation of cAMP metabolic process	GO:0030816	0.286	1
cellular response to glucose stimulus	GO:0071333	0.286	1
artery development	GO:0060840	0.286	1
positive regulation of signaling	GO:0023056	0.286	6
immune system development	GO:0002520	0.287	4
T cell mediated immunity	GO:0002456	0.288	1
response to growth factor	GO:0070848	0.288	3
positive regulation of cell communication	GO:0010647	0.288	6

regulation of fibril organization	GO:1902903	0.288	2
cellular response to hexose stimulus	GO:0071331	0.288	1
cellular response to monosaccharide stimulus	GO:0071326	0.288	1
regulation of hemostasis	GO:1900046	0.288	1
vesicle-mediated transport to the plasma membrane	GO:0098876	0.288	1
regulation of blood coagulation	GO:0030193	0.288	1
positive regulation of production of molecular mediator of immune response	GO:0002702	0.288	1
cell-cell adhesion	GO:0098609	0.289	4
actin filament-based process	GO:0030029	0.289	4
secretion by cell	GO:0032940	0.289	4
positive regulation of ossification	GO:0045778	0.289	1
positive regulation of calcium ion transport	GO:0051928	0.289	1
positive regulation of myeloid cell differentiation	GO:0045639	0.289	1
regulation of response to wounding	GO:1903034	0.29	2
cellular component organization	GO:0016043	0.29	21
positive regulation of cell-substrate adhesion	GO:0010811	0.291	1
carbohydrate derivative transport	GO:1901264	0.291	1
regulation of cell-matrix adhesion	GO:0001952	0.291	1
positive regulation of cell adhesion	GO:0045785	0.291	2
regulation of protein metabolic process	GO:0051246	0.291	10
axis specification	GO:0009798	0.293	1
regulation of secretion by cell	GO:1903530	0.294	3
intracellular protein transport	GO:0006886	0.294	5
female gonad development	GO:0008585	0.294	1
regulation of coagulation	GO:0050818	0.294	1
cytokine production involved in immune response	GO:0002367	0.294	1

head development	GO:0060322	0.295	3
positive regulation of adaptive immune response based on somatic recombination of immune receptors built from immunoglobulin superfamily domains	GO:0002824	0.295	1
spinal cord development	GO:0021510	0.295	1
negative regulation of leukocyte differentiation	GO:1902106	0.295	1
positive regulation of cyclic nucleotide biosynthetic process	GO:0030804	0.295	1
palate development	GO:0060021	0.295	1
negative regulation of cell morphogenesis involved in differentiation	GO:0010771	0.295	1
development of primary female sexual characteristics	GO:0046545	0.295	1
regulation of multicellular organism growth	GO:0040014	0.295	1
lipid catabolic process	GO:0016042	0.295	2
regulation of cytokinesis	GO:0032465	0.295	1
lysosomal transport	GO:0007041	0.295	1
cellular response to hypoxia	GO:0071456	0.295	1
negative regulation of transmembrane transport	GO:0034763	0.295	1
cellular response to decreased oxygen levels	GO:0036294	0.295	1
negative regulation of cellular protein catabolic process	GO:1903363	0.295	1
epithelial cell proliferation	GO:0050673	0.295	2
regulation of transforming growth factor beta receptor signaling pathway	GO:0017015	0.297	1
intrinsic apoptotic signaling pathway by p53 class mediator	GO:0072332	0.297	1
negative regulation of protein secretion	GO:0050709	0.297	1
regulation of striated muscle cell differentiation	GO:0051153	0.297	1
presynaptic process involved in chemical synaptic transmission	GO:0099531	0.297	1
protein polymerization	GO:0051258	0.298	2
adherens junction organization	GO:0034332	0.298	1
regulation of cellular response to transforming growth factor beta stimulus	GO:1903844	0.298	1

centrosome duplication	GO:0051298	0.298	1
positive regulation of proteasomal ubiquitin-dependent protein catabolic process	GO:0032436	0.298	1
alpha-beta T cell differentiation	GO:0046632	0.298	1
regulation of nitrogen compound metabolic process	GO:0051171	0.298	17
immune response-activating signal transduction	GO:0002757	0.299	2
RNA phosphodiester bond hydrolysis, endonucleolytic	GO:0090502	0.299	1
cellular response to carbohydrate stimulus	GO:0071322	0.299	1
cell aging	GO:0007569	0.299	1
DNA replication	GO:0006260	0.3	2
eye development	GO:0001654	0.3	2
establishment of protein localization	GO:0045184	0.3	7
regulation of stem cell proliferation	GO:0072091	0.3	1
positive regulation of adaptive immune response	GO:0002821	0.3	1
kidney morphogenesis	GO:0060993	0.3	1
RNA methylation	GO:0001510	0.3	1
positive regulation of T cell proliferation	GO:0042102	0.302	1
catabolic process	GO:0009056	0.303	9
regulation of protein secretion	GO:0050708	0.303	2
localization	GO:0051179	0.303	22
regulation of synapse assembly	GO:0051963	0.304	1
regulation of extent of cell growth	GO:0061387	0.304	1
behavior	GO:0007610	0.305	3
hair cycle process	GO:0022405	0.305	1
centrosome organization	GO:0051297	0.305	1
positive regulation of cyclic nucleotide metabolic process	GO:0030801	0.305	1
molting cycle process	GO:0022404	0.305	1

regulation of cyclase activity	GO:0031279	0.307	1
nucleobase-containing small molecule metabolic process	GO:0055086	0.307	4
purine ribonucleotide metabolic process	GO:0009150	0.308	3
regulation of dendrite development	GO:0050773	0.308	1
regulation of circadian rhythm	GO:0042752	0.308	1
somite development	GO:0061053	0.308	1
maintenance of location	GO:0051235	0.308	2
macromolecule localization	GO:0033036	0.309	10
regulation of lyase activity	GO:0051339	0.309	1
positive regulation of ion transmembrane transport	GO:0034767	0.309	1
determination of left/right symmetry	GO:0007368	0.309	1
positive regulation of lymphocyte mediated immunity	GO:0002708	0.309	1
positive regulation of synaptic transmission	GO:0050806	0.311	1
amino acid transmembrane transport	GO:0003333	0.311	1
cell migration	GO:0016477	0.313	5
positive regulation of translation	GO:0045727	0.313	1
negative regulation of neuron projection development	GO:0010977	0.316	1
single-organism membrane organization	GO:0044802	0.316	3
regulation of secretion	GO:0051046	0.316	3
immune response-regulating signaling pathway	GO:0002764	0.316	2
apoptotic process	GO:0006915	0.317	6
regulation of G1/S transition of mitotic cell cycle	GO:2000045	0.317	1
cellular calcium ion homeostasis	GO:0006874	0.317	2
ribonucleotide metabolic process	GO:0009259	0.317	3
regionalization	GO:0003002	0.318	2
cellular response to oxygen levels	GO:0071453	0.318	1

detection of abiotic stimulus	GO:0009582	0.318	1
ureteric bud development	GO:0001657	0.318	1
mesonephric epithelium development	GO:0072163	0.32	1
mesonephric tubule development	GO:0072164	0.32	1
gland development	GO:0048732	0.32	2
positive regulation of nucleotide biosynthetic process	GO:0030810	0.32	1
inner ear morphogenesis	GO:0042472	0.32	1
positive regulation of purine nucleotide biosynthetic process	GO:1900373	0.32	1
regulation of myeloid leukocyte differentiation	GO:0002761	0.32	1
regulation of cAMP biosynthetic process	GO:0030817	0.32	1
purine nucleotide metabolic process	GO:0006163	0.321	3
detection of external stimulus	GO:0009581	0.321	1
protein export from nucleus	GO:0006611	0.321	1
determination of bilateral symmetry	GO:0009855	0.321	1
female sex differentiation	GO:0046660	0.321	1
positive regulation of epithelial cell migration	GO:0010634	0.321	1
phospholipase C-activating G-protein coupled receptor signaling pathway	GO:0007200	0.321	1
pigmentation	GO:0043473	0.321	1
specification of symmetry	GO:0009799	0.323	1
response to organonitrogen compound	GO:0010243	0.323	3
programmed cell death	GO:0012501	0.323	6
establishment of localization	GO:0051234	0.323	18
regulation of chromosome segregation	GO:0051983	0.324	1
neural tube closure	GO:0001843	0.324	1
calcium ion homeostasis	GO:0055074	0.324	2
sequestering of metal ion	GO:0051238	0.325	1

cellular glucose homeostasis	GO:0001678	0.325	1
negative regulation of ion transport	GO:0043271	0.325	1
tube closure	GO:0060606	0.325	1
calcium ion transmembrane import into cytosol	GO:0097553	0.325	1
regulation of stem cell differentiation	GO:2000736	0.325	1
ribose phosphate metabolic process	GO:0019693	0.325	3
purine ribonucleotide biosynthetic process	GO:0009152	0.325	2
adaptive immune response	GO:0002250	0.325	2
positive regulation of peptide secretion	GO:0002793	0.325	1
positive regulation of proteasomal protein catabolic process	GO:1901800	0.325	1
cardiac muscle contraction	GO:0060048	0.325	1
regulation of striated muscle tissue development	GO:0016202	0.327	1
mesonephros development	GO:0001823	0.327	1
release of sequestered calcium ion into cytosol	GO:0051209	0.327	1
positive regulation of catabolic process	GO:0009896	0.328	2
cell-cell signaling by wnt	GO:0198738	0.328	2
regulation of cation channel activity	GO:2001257	0.328	1
negative regulation of sequestering of calcium ion	GO:0051283	0.328	1
regulation of cell cycle G1/S phase transition	GO:1902806	0.328	1
neuron-neuron synaptic transmission	GO:0007270	0.328	1
regulation of organelle organization	GO:0033043	0.328	5
carboxylic acid biosynthetic process	GO:0046394	0.328	2
organic acid biosynthetic process	GO:0016053	0.328	2
muscle cell differentiation	GO:0042692	0.328	2
natural killer cell activation	GO:0030101	0.328	1
response to glucose	GO:0009749	0.328	1

regulation of muscle organ development	GO:0048634	0.328	1
inorganic cation transmembrane transport	GO:0098662	0.328	4
cation homeostasis	GO:0055080	0.328	3
nucleoside triphosphate metabolic process	GO:0009141	0.328	2
primary neural tube formation	GO:0014020	0.328	1
regulation of muscle tissue development	GO:1901861	0.328	1
molting cycle	GO:0042303	0.328	1
post-Golgi vesicle-mediated transport	GO:0006892	0.328	1
camera-type eye morphogenesis	GO:0048593	0.328	1
hair cycle	GO:0042633	0.328	1
regulation of sequestering of calcium ion	GO:0051282	0.328	1
sequestering of calcium ion	GO:0051208	0.328	1
regulation of BMP signaling pathway	GO:0030510	0.328	1
regulation of cell migration	GO:0030334	0.328	3
calcium ion transmembrane transport	GO:0070588	0.328	2
cell cycle	GO:0007049	0.329	6
response to hexose	GO:0009746	0.329	1
response to monosaccharide	GO:0034284	0.329	1
regulation of transferase activity	GO:0051338	0.329	4
alpha-amino acid biosynthetic process	GO:1901607	0.33	1
ribonucleotide biosynthetic process	GO:0009260	0.332	2
purine nucleotide biosynthetic process	GO:0006164	0.332	2
regulation of apoptotic signaling pathway	GO:2001233	0.332	2
ribose phosphate biosynthetic process	GO:0046390	0.333	2
positive regulation of cellular amide metabolic process	GO:0034250	0.333	1
positive regulation of leukocyte mediated immunity	GO:0002705	0.333	1

positive regulation of transmembrane transport	GO:0034764	0.335	1
endocrine system development	GO:0035270	0.335	1
mesoderm development	GO:0007498	0.335	1
inorganic ion homeostasis	GO:0098771	0.335	3
erythrocyte differentiation	GO:0030218	0.336	1
learning	GO:0007612	0.336	1
alpha-beta T cell activation	GO:0046631	0.336	1
regulation of apoptotic process	GO:0042981	0.337	5
regulation of cellular carbohydrate metabolic process	GO:0010675	0.338	1
regulation of cyclic nucleotide biosynthetic process	GO:0030802	0.338	1
nucleus organization	GO:0006997	0.339	1
regulation of blood vessel size	GO:0050880	0.339	1
regulation of cAMP metabolic process	GO:0030814	0.339	1
positive regulation of cell growth	GO:0030307	0.339	1
cell cycle phase transition	GO:0044770	0.339	2
positive regulation of protein ubiquitination	GO:0031398	0.34	1
myotube differentiation	GO:0014902	0.34	1
epithelial to mesenchymal transition	GO:0001837	0.34	1
regulation of programmed cell death	GO:0043067	0.34	5
secretion	GO:0046903	0.34	4
positive regulation of response to wounding	GO:1903036	0.34	1
regulation of receptor activity	GO:0010469	0.34	1
regulation of fat cell differentiation	GO:0045598	0.34	1
cellular response to metal ion	GO:0071248	0.34	1
regulation of tube size	GO:0035150	0.34	1
purine-containing compound biosynthetic process	GO:0072522	0.34	2

cellular divalent inorganic cation homeostasis	GO:0072503	0.34	2
positive regulation of nucleotide metabolic process	GO:0045981	0.34	1
regulation of production of molecular mediator of immune response	GO:0002700	0.34	1
positive regulation of purine nucleotide metabolic process	GO:1900544	0.34	1
positive regulation of proteolysis involved in cellular protein catabolic process	GO:1903052	0.34	1
chondrocyte differentiation	GO:0002062	0.34	1
regulation of ion homeostasis	GO:2000021	0.34	1
negative regulation of canonical Wnt signaling pathway	GO:0090090	0.34	1
purine-containing compound metabolic process	GO:0072521	0.341	3
carboxylic acid transmembrane transport	GO:1905039	0.341	1
positive regulation of homeostatic process	GO:0032846	0.341	1
regulation of insulin secretion	GO:0050796	0.341	1
regulation of epithelial cell differentiation	GO:0030856	0.341	1
regulation of cellular ketone metabolic process	GO:0010565	0.341	1
activation of immune response	GO:0002253	0.341	2
negative regulation of cell projection organization	GO:0031345	0.342	1
cellular amino acid biosynthetic process	GO:0008652	0.342	1
regulation of cellular localization	GO:0060341	0.343	4
cellular response to organonitrogen compound	GO:0071417	0.343	2
response to hormone	GO:0009725	0.343	3
positive regulation of transmembrane receptor protein serine/threonine kinase signaling pathway	GO:0090100	0.343	1
positive regulation of lymphocyte proliferation	GO:0050671	0.343	1
negative regulation of hemopoiesis	GO:1903707	0.343	1
negative regulation of transport	GO:0051051	0.343	2
response to carbohydrate	GO:0009743	0.344	1
positive regulation of mononuclear cell proliferation	GO:0032946	0.344	1

positive regulation of cell division	GO:0051781	0.344	1
regulation of cellular catabolic process	GO:0031329	0.344	3
negative regulation of homeostatic process	GO:0032845	0.346	1
regulation of cell motility	GO:2000145	0.347	3
positive regulation of lipid metabolic process	GO:0045834	0.347	1
erythrocyte homeostasis	GO:0034101	0.347	1
cellular component assembly	GO:0022607	0.35	9
sulfur compound biosynthetic process	GO:0044272	0.35	1
calcium ion transport into cytosol	GO:0060402	0.35	1
protein targeting to membrane	GO:0006612	0.35	1
cell-cell signaling	GO:0007267	0.35	5
positive regulation of leukocyte proliferation	GO:0070665	0.351	1
formation of primary germ layer	GO:0001704	0.351	1
positive regulation of leukocyte differentiation	GO:1902107	0.351	1
localization of cell	GO:0051674	0.351	5
cell motility	GO:0048870	0.351	5
divalent inorganic cation homeostasis	GO:0072507	0.351	2
mammary gland development	GO:0030879	0.352	1
cell projection assembly	GO:0030031	0.352	2
cell surface receptor signaling pathway	GO:0007166	0.353	9
sensory perception of sound	GO:0007605	0.355	1
multicellular organismal signaling	GO:0035637	0.357	1
positive regulation of transport	GO:0051050	0.358	3
Wnt signaling pathway	GO:0016055	0.358	2
cilium assembly	GO:0042384	0.358	1
female gamete generation	GO:0007292	0.358	1

regulation of biological process	GO:0050789	0.358	42
transport	GO:0006810	0.36	17
negative regulation of cellular protein localization	GO:1903828	0.36	1
positive regulation of protein modification by small protein conjugation or removal	GO:1903322	0.36	1
regulation of adaptive immune response based on somatic recombination of immune receptors built from immunoglobulin superfamily domains	GO:0002822	0.36	1
aging	GO:0007568	0.36	1
positive regulation of cellular protein catabolic process	GO:1903364	0.36	1
regulation of lymphocyte mediated immunity	GO:0002706	0.362	1
regulation of cyclic nucleotide metabolic process	GO:0030799	0.362	1
regulation of muscle contraction	GO:0006937	0.362	1
monovalent inorganic cation transport	GO:0015672	0.362	3
nucleoside diphosphate phosphorylation	GO:0006165	0.363	1
ion homeostasis	GO:0050801	0.364	3
response to chemical	GO:0042221	0.364	16
regulation of protein phosphorylation	GO:0001932	0.364	5
cellular response to radiation	GO:0071478	0.364	1
vascular process in circulatory system	GO:0003018	0.364	1
negative regulation of response to wounding	GO:1903035	0.364	1
cytosolic calcium ion transport	GO:0060401	0.365	1
regulation of carbohydrate metabolic process	GO:0006109	0.365	1
positive regulation of homotypic cell-cell adhesion	GO:0034112	0.367	1
negative regulation of apoptotic process	GO:0043066	0.367	3
negative regulation of secretion by cell	GO:1903531	0.368	1
negative regulation of protein catabolic process	GO:0042177	0.368	1
macromolecular complex assembly	GO:0065003	0.369	6

regulation of cellular process	GO:0050794	0.369	40
calcium ion import	GO:0070509	0.369	1
fatty acid biosynthetic process	GO:0006633	0.369	1
regulation of synapse organization	GO:0050807	0.369	1
visual perception	GO:0007601	0.37	1
adenylate cyclase-activating G-protein coupled receptor signaling pathway	GO:0007189	0.372	1
phosphate-containing compound metabolic process	GO:0006796	0.372	13
negative regulation of programmed cell death	GO:0043069	0.372	3
peptide secretion	GO:0002790	0.372	2
negative regulation of cell growth	GO:0030308	0.372	1
signal transduction by p53 class mediator	GO:0072331	0.372	1
protein phosphorylation	GO:0006468	0.373	8
pattern specification process	GO:0007389	0.375	2
integrin-mediated signaling pathway	GO:0007229	0.375	1
regulation of muscle cell differentiation	GO:0051147	0.375	1
retina development in camera-type eye	GO:0060041	0.375	1
positive regulation of cell morphogenesis involved in differentiation	GO:0010770	0.375	1
regulation of intracellular signal transduction	GO:1902531	0.375	6
limb morphogenesis	GO:0035108	0.376	1
sensory perception of mechanical stimulus	GO:0050954	0.376	1
appendage morphogenesis	GO:0035107	0.376	1
regulation of phosphate metabolic process	GO:0019220	0.376	6
protein secretion	GO:0009306	0.376	2
myeloid leukocyte activation	GO:0002274	0.376	1
signal release from synapse	GO:0099643	0.376	1
regulation of phosphorus metabolic process	GO:0051174	0.377	6

myeloid cell homeostasis	GO:0002262	0.377	1
cell recognition	GO:0008037	0.377	1
DNA damage checkpoint	GO:0000077	0.377	1
organic acid transmembrane transport	GO:1903825	0.377	1
regulation of peptide hormone secretion	GO:0090276	0.377	1
sensory perception of light stimulus	GO:0050953	0.377	1
regulation of cell projection assembly	GO:0060491	0.377	1
cell cycle arrest	GO:0007050	0.377	1
regulation of nucleotide biosynthetic process	GO:0030808	0.377	1
regulation of STAT cascade	GO:1904892	0.377	1
organ growth	GO:0035265	0.377	1
regulation of purine nucleotide biosynthetic process	GO:1900371	0.377	1
phosphorus metabolic process	GO:0006793	0.377	13
eye morphogenesis	GO:0048592	0.378	1
regulation of cell shape	GO:0008360	0.378	1
regulation of T cell proliferation	GO:0042129	0.378	1
positive regulation of epithelial cell proliferation	GO:0050679	0.378	1
cellular nitrogen compound catabolic process	GO:0044270	0.378	2
single-organism nuclear import	GO:1902593	0.379	1
regulation of locomotion	GO:0040012	0.379	3
response to other organism	GO:0051707	0.38	3
regulation of lymphocyte differentiation	GO:0045619	0.38	1
amino acid transport	GO:0006865	0.38	1
nucleotide phosphorylation	GO:0046939	0.38	1
synapse assembly	GO:0007416	0.38	1
response to external biotic stimulus	GO:0043207	0.38	3

cation transmembrane transport	GO:0098655	0.38	4
inorganic ion transmembrane transport	GO:0098660	0.38	4
heterocycle catabolic process	GO:0046700	0.38	2
regulation of cellular component movement	GO:0051270	0.38	3
negative regulation of establishment of protein localization	GO:1904950	0.381	1
peptidyl-amino acid modification	GO:0018193	0.381	5
stem cell population maintenance	GO:0019827	0.383	1
positive regulation of cellular protein metabolic process	GO:0032270	0.383	5
negative regulation of secretion	GO:0051048	0.384	1
maintenance of cell number	GO:0098727	0.385	1
striated muscle contraction	GO:0006941	0.385	1
response to nitrogen compound	GO:1901698	0.385	3
negative regulation of cell cycle phase transition	GO:1901988	0.386	1
regulation of blood pressure	GO:0008217	0.386	1
DNA integrity checkpoint	GO:0031570	0.386	1
insulin secretion	GO:0030073	0.386	1
negative regulation of Wnt signaling pathway	GO:0030178	0.387	1
ribosomal large subunit biogenesis	GO:0042273	0.389	1
positive regulation of developmental growth	GO:0048639	0.39	1
regulation of heart contraction	GO:0008016	0.392	1
protein stabilization	GO:0050821	0.393	1
regulation of response to stimulus	GO:0048583	0.394	11
cellular response to nitrogen compound	GO:1901699	0.394	2
centrosome cycle	GO:0007098	0.394	1
cation transport	GO:0006812	0.394	5
vacuolar transport	GO:0007034	0.395	1

regulation of peptidyl-serine phosphorylation	GO:0033135	0.397	1
nucleoside triphosphate biosynthetic process	GO:0009142	0.398	1
cell surface receptor signaling pathway involved in cell-cell signaling	GO:1905114	0.399	2
actin filament organization	GO:0007015	0.4	2
neurotransmitter secretion	GO:0007269	0.401	1
cellular protein localization	GO:0034613	0.402	6
positive regulation of hemopoiesis	GO:1903708	0.402	1
regulation of proteolysis	GO:0030162	0.403	3
limb development	GO:0060173	0.403	1
negative regulation of translation	GO:0017148	0.403	1
appendage development	GO:0048736	0.403	1
response to biotic stimulus	GO:0009607	0.404	3
positive regulation of T cell activation	GO:0050870	0.404	1
cellular macromolecule localization	GO:0070727	0.405	6
STAT cascade	GO:0097696	0.405	1
blood vessel morphogenesis	GO:0048514	0.405	2
neuron migration	GO:0001764	0.406	1
response to hypoxia	GO:0001666	0.406	1
response to decreased oxygen levels	GO:0036293	0.406	1
cellular catabolic process	GO:0044248	0.406	7
response to stimulus	GO:0050896	0.406	31
cell junction assembly	GO:0034329	0.406	1
vesicle fusion	GO:0006906	0.406	1
nucleoside diphosphate metabolic process	GO:0009132	0.406	1
cell-cell junction organization	GO:0045216	0.407	1
skeletal muscle tissue development	GO:0007519	0.407	1

regulation of phosphorylation	GO:0042325	0.407	5
organic cyclic compound catabolic process	GO:1901361	0.408	2
cytokinesis	GO:0000910	0.408	1
cellular potassium ion transport	GO:0071804	0.41	1
skeletal system development	GO:0001501	0.41	2
regulation of myeloid cell differentiation	GO:0045637	0.41	1
regulation of JAK-STAT cascade	GO:0046425	0.41	1
gonad development	GO:0008406	0.41	1
development of primary sexual characteristics	GO:0045137	0.412	1
positive regulation of ion transport	GO:0043270	0.412	1
regulation of cell size	GO:0008361	0.412	1
regulation of leukocyte mediated immunity	GO:0002703	0.412	1
negative regulation of protein transport	GO:0051224	0.412	1
positive regulation of leukocyte cell-cell adhesion	GO:1903039	0.412	1
cell maturation	GO:0048469	0.412	1
regulation of muscle system process	GO:0090257	0.412	1
organelle assembly	GO:0070925	0.412	3
response to bacterium	GO:0009617	0.412	2
hormone metabolic process	GO:0042445	0.412	1
microtubule organizing center organization	GO:0031023	0.412	1
G1/S transition of mitotic cell cycle	GO:0000082	0.413	1
branching morphogenesis of an epithelial tube	GO:0048754	0.413	1
skeletal muscle organ development	GO:0060538	0.413	1
regulation of defense response	GO:0031347	0.414	2
ion transmembrane transport	GO:0034220	0.414	5
regulation of organ morphogenesis	GO:2000027	0.414	1

positive regulation of apoptotic process	GO:0043065	0.416	2
gamete generation	GO:0007276	0.416	2
developmental cell growth	GO:0048588	0.416	1
positive regulation of programmed cell death	GO:0043068	0.417	2
positive regulation of immune effector process	GO:0002699	0.417	1
positive regulation of molecular function	GO:0044093	0.418	6
multicellular organism growth	GO:0035264	0.418	1
response to oxygen levels	GO:0070482	0.418	1
proteolysis	GO:0006508	0.419	7
positive regulation of I-kappaB kinase/NF-kappaB signaling	GO:0043123	0.419	1
growth	GO:0040007	0.42	3
negative regulation of cellular amide metabolic process	GO:0034249	0.42	1
learning or memory	GO:0007611	0.421	1
positive regulation of protein secretion	GO:0050714	0.421	1
small molecule metabolic process	GO:0044281	0.422	7
positive regulation of protein metabolic process	GO:0051247	0.422	5
cellular macromolecular complex assembly	GO:0034622	0.422	4
cellular metal ion homeostasis	GO:0006875	0.422	2
gastrulation	GO:0007369	0.422	1
T cell proliferation	GO:0042098	0.422	1
inner ear development	GO:0048839	0.422	1
cell cycle G1/S phase transition	GO:0044843	0.422	1
sodium ion transmembrane transport	GO:0035725	0.422	1
positive regulation of response to stimulus	GO:0048584	0.423	6
response to metal ion	GO:0010038	0.423	1
transition metal ion homeostasis	GO:0055076	0.425	1

proteasome-mediated ubiquitin-dependent protein catabolic process	GO:0043161	0.425	2
regulation of response to DNA damage stimulus	GO:2001020	0.429	1
negative regulation of cellular catabolic process	GO:0031330	0.429	1
cellular ketone metabolic process	GO:0042180	0.43	1
myeloid leukocyte differentiation	GO:0002573	0.43	1
cellular response to stress	GO:0033554	0.432	6
peptide hormone secretion	GO:0030072	0.432	1
phosphorylation	GO:0016310	0.432	9
regulation of ion transmembrane transporter activity	GO:0032412	0.433	1
positive regulation of cell death	GO:0010942	0.434	2
glucose homeostasis	GO:0042593	0.434	1
tissue homeostasis	GO:0001894	0.434	1
blood coagulation	GO:0007596	0.434	1
lung development	GO:0030324	0.434	1
cartilage development	GO:0051216	0.434	1
carbohydrate homeostasis	GO:0033500	0.434	1
endomembrane system organization	GO:0010256	0.435	2
hemostasis	GO:0007599	0.435	1
heart contraction	GO:0060047	0.435	1
organic substance transport	GO:0071702	0.436	8
actomyosin structure organization	GO:0031032	0.436	1
coagulation	GO:0050817	0.436	1
nuclear export	GO:0051168	0.436	1
respiratory tube development	GO:0030323	0.436	1
regulation of transmembrane transporter activity	GO:0022898	0.436	1
negative regulation of cell proliferation	GO:0008285	0.439	2

dendrite development	GO:0016358	0.439	1
coenzyme biosynthetic process	GO:0009108	0.44	1
RNA phosphodiester bond hydrolysis	GO:0090501	0.44	1
heart process	GO:0003015	0.441	1
JAK-STAT cascade	GO:0007259	0.442	1
regulation of purine nucleotide metabolic process	GO:1900542	0.443	1
positive regulation of cell-cell adhesion	GO:0022409	0.443	1
single-organism metabolic process	GO:0044710	0.444	11
carbohydrate derivative biosynthetic process	GO:1901137	0.444	3
regulation of blood circulation	GO:1903522	0.445	1
RNA modification	GO:0009451	0.445	1
spindle organization	GO:0007051	0.446	1
regulation of nucleotide metabolic process	GO:0006140	0.447	1
regulation of synapse structure or activity	GO:0050803	0.447	1
nuclear import	GO:0051170	0.447	1
nucleobase-containing compound transport	GO:0015931	0.45	1
positive regulation of immune response	GO:0050778	0.451	2
proteasomal protein catabolic process	GO:0010498	0.451	2
regulation of transporter activity	GO:0032409	0.453	1
positive regulation of protein catabolic process	GO:0045732	0.454	1
fat cell differentiation	GO:0045444	0.455	1
homophilic cell adhesion via plasma membrane adhesion molecules	GO:0007156	0.455	1
cognition	GO:0050890	0.456	1
morphogenesis of a branching epithelium	GO:0061138	0.456	1
homeostatic process	GO:0042592	0.456	5
organelle localization	GO:0051640	0.456	2

response to stress	GO:0006950	0.457	10
regulation of peptide secretion	GO:0002791	0.457	1
protein polyubiquitination	GO:0000209	0.457	1
regulation of homotypic cell-cell adhesion	GO:0034110	0.458	1
cell junction organization	GO:0034330	0.458	1
regulation of canonical Wnt signaling pathway	GO:0060828	0.459	1
positive regulation of cytosolic calcium ion concentration	GO:0007204	0.46	1
skin development	GO:0043588	0.46	1
cytoskeleton organization	GO:0007010	0.46	5
respiratory system development	GO:0060541	0.464	1
regulation of signal transduction	GO:0009966	0.464	8
positive regulation of growth	GO:0045927	0.465	1
epithelial cell development	GO:0002064	0.466	1
blood vessel development	GO:0001568	0.466	2
monocarboxylic acid metabolic process	GO:0032787	0.469	2
RNA catabolic process	GO:0006401	0.472	1
cellular response to abiotic stimulus	GO:0071214	0.474	1
monocarboxylic acid biosynthetic process	GO:0072330	0.474	1
positive regulation of sequence-specific DNA binding transcription factor activity	GO:0051091	0.475	1
sex differentiation	GO:0007548	0.475	1
morphogenesis of a branching structure	GO:0001763	0.475	1
peptidyl-tyrosine dephosphorylation	GO:0035335	0.475	1
anterior/posterior pattern specification	GO:0009952	0.475	1
adenylate cyclase-modulating G-protein coupled receptor signaling pathway	GO:0007188	0.478	1
microtubule-based process	GO:0007017	0.478	3
anterograde trans-synaptic signaling	GO:0098916	0.479	2

developmental growth involved in morphogenesis	GO:0060560	0.481	1
cellular cation homeostasis	GO:0030003	0.481	2
defense response to virus	GO:0051607	0.481	1
trans-synaptic signaling	GO:0099537	0.481	2
negative regulation of catabolic process	GO:0009895	0.483	1
apoptotic signaling pathway	GO:0097190	0.483	2
multicellular organismal reproductive process	GO:0048609	0.483	2
taxis	GO:0042330	0.483	2
chemotaxis	GO:0006935	0.483	2
neurotransmitter transport	GO:0006836	0.484	1
vasculature development	GO:0001944	0.484	2
negative regulation of cell cycle process	GO:0010948	0.485	1
regulation of I-kappaB kinase/NF-kappaB signaling	GO:0043122	0.485	1
synaptic signaling	GO:0099536	0.486	2
membrane organization	GO:0061024	0.486	3
multi-organism process	GO:0051704	0.486	5
regulation of transmembrane receptor protein serine/threonine kinase signaling pathway	GO:0090092	0.487	1
organophosphate metabolic process	GO:0019637	0.49	4
cellular ion homeostasis	GO:0006873	0.491	2
regulation of Rho protein signal transduction	GO:0035023	0.491	1
regulation of leukocyte differentiation	GO:1902105	0.491	1
negative regulation of protein kinase activity	GO:0006469	0.492	1
obsolete cell-type specific apoptotic process	GO:0097285	0.493	1
regulation of protein transport	GO:0051223	0.494	2
regulation of protein stability	GO:0031647	0.494	1
multicellular organism reproduction	GO:0032504	0.495	2

lymphocyte mediated immunity	GO:0002449	0.497	1
regulation of cytosolic calcium ion concentration	GO:0051480	0.497	1
small molecule biosynthetic process	GO:0044283	0.497	2
lipid metabolic process	GO:0006629	0.499	4
purine ribonucleoside metabolic process	GO:0046128	0.499	1
leukocyte activation involved in immune response	GO:0002366	0.499	1
neuron death	GO:0070997	0.5	1
cellular response to hormone stimulus	GO:0032870	0.502	2
G-protein coupled receptor signaling pathway, coupled to cyclic nucleotide second messenger	GO:0007187	0.502	1
purine nucleoside metabolic process	GO:0042278	0.502	1
cell activation involved in immune response	GO:0002263	0.503	1
adaptive immune response based on somatic recombination of immune receptors built from immunoglobulin superfamily domains	GO:0002460	0.504	1
negative regulation of response to external stimulus	GO:0032102	0.504	1
negative regulation of kinase activity	GO:0033673	0.504	1
monosaccharide metabolic process	GO:0005996	0.504	1
cofactor biosynthetic process	GO:0051188	0.504	1
regulation of mitotic cell cycle phase transition	GO:1901990	0.505	1
regulation of cellular response to growth factor stimulus	GO:0090287	0.506	1
production of molecular mediator of immune response	GO:0002440	0.509	1
positive regulation of transferase activity	GO:0051347	0.51	2
innate immune response	GO:0045087	0.511	2
cellular response to stimulus	GO:0051716	0.511	26
regulation of neurotransmitter levels	GO:0001505	0.511	1
positive regulation of lymphocyte activation	GO:0051251	0.511	1
protein homooligomerization	GO:0051260	0.515	1

regulation of T cell activation	GO:0050863	0.516	1
sexual reproduction	GO:0019953	0.517	2
ion transport	GO:0006811	0.517	6
ribonucleoside metabolic process	GO:0009119	0.517	1
mitochondrial transport	GO:0006839	0.517	1
homeostasis of number of cells	GO:0048872	0.517	1
regulation of cytoplasmic transport	GO:1903649	0.517	1
regulation of gene expression, epigenetic	GO:0040029	0.518	1
regulation of leukocyte cell-cell adhesion	GO:1903037	0.518	1
cellular response to steroid hormone stimulus	GO:0071383	0.519	1
carbohydrate derivative metabolic process	GO:1901135	0.519	4
immune effector process	GO:0002252	0.519	2
chemical homeostasis	GO:0048878	0.519	3
potassium ion transmembrane transport	GO:0071805	0.519	1
I-kappaB kinase/NF-kappaB signaling	GO:0007249	0.519	1
regulation of cellular response to stress	GO:0080135	0.519	2
response to organic cyclic compound	GO:0014070	0.521	2
biological regulation	GO:0065007	0.523	42
cell-cell adhesion via plasma-membrane adhesion molecules	GO:0098742	0.523	1
regulation of establishment of protein localization	GO:0070201	0.524	2
regulation of epithelial cell proliferation	GO:0050678	0.527	1
alpha-amino acid metabolic process	GO:1901605	0.531	1
muscle contraction	GO:0006936	0.531	1
regulation of cell division	GO:0051302	0.531	1
transmembrane receptor protein tyrosine kinase signaling pathway	GO:0007169	0.532	2
negative regulation of signal transduction	GO:0009968	0.533	3

canonical Wnt signaling pathway	GO:0060070	0.536	1
establishment of protein localization to membrane	GO:0090150	0.536	1
immune response	GO:0006955	0.538	4
positive regulation of leukocyte activation	GO:0002696	0.539	1
chemical synaptic transmission	GO:0007268	0.539	2
cilium organization	GO:0044782	0.539	1
regulation of Wnt signaling pathway	GO:0030111	0.539	1
microtubule cytoskeleton organization	GO:0000226	0.543	2
positive regulation of cell activation	GO:0050867	0.546	1
response to virus	GO:0009615	0.548	1
regulation of peptide transport	GO:0090087	0.548	1
nuclear chromosome segregation	GO:0098813	0.549	1
DNA metabolic process	GO:0006259	0.549	3
regulation of catalytic activity	GO:0050790	0.55	7
nucleoside metabolic process	GO:0009116	0.55	1
modulation of synaptic transmission	GO:0050804	0.552	1
Rho protein signal transduction	GO:0007266	0.553	1
carboxylic acid metabolic process	GO:0019752	0.56	3
intracellular signal transduction	GO:0035556	0.562	8
leukocyte mediated immunity	GO:0002443	0.563	1
response to steroid hormone	GO:0048545	0.563	1
positive regulation of cellular catabolic process	GO:0031331	0.563	1
regulation of cell growth	GO:0001558	0.565	1
cellular chemical homeostasis	GO:0055082	0.567	2
regulation of immune effector process	GO:0002697	0.57	1
potassium ion transport	GO:0006813	0.57	1

sulfur compound metabolic process	GO:0006790	0.57	1
glycosyl compound metabolic process	GO:1901657	0.572	1
positive regulation of phosphorus metabolic process	GO:0010562	0.572	3
positive regulation of phosphate metabolic process	GO:0045937	0.572	3
microtubule-based movement	GO:0007018	0.572	1
regulation of hemopoiesis	GO:1903706	0.572	1
positive regulation of proteolysis	GO:0045862	0.572	1
negative regulation of signaling	GO:0023057	0.575	3
regulation of signaling	GO:0023051	0.575	8
multi-organism reproductive process	GO:0044703	0.575	2
negative regulation of cell communication	GO:0010648	0.575	3
cilium morphogenesis	GO:0060271	0.577	1
multicellular organismal homeostasis	GO:0048871	0.578	1
carboxylic acid transport	GO:0046942	0.579	1
Golgi vesicle transport	GO:0048193	0.58	1
single organism reproductive process	GO:0044702	0.582	2
single-organism catabolic process	GO:0044712	0.585	2
muscle system process	GO:0003012	0.585	1
organic acid transport	GO:0015849	0.585	1
single-organism cellular localization	GO:1902580	0.586	2
oxoacid metabolic process	GO:0043436	0.588	3
anatomical structure homeostasis	GO:0060249	0.588	1
regulation of chromosome organization	GO:0033044	0.588	1
myeloid cell differentiation	GO:0030099	0.588	1
regulation of cell communication	GO:0010646	0.588	8
positive regulation of protein modification process	GO:0031401	0.588	3

cellular carbohydrate metabolic process	GO:0044262	0.592	1
purine ribonucleoside triphosphate metabolic process	GO:0009205	0.592	1
regulation of Ras protein signal transduction	GO:0046578	0.593	1
positive regulation of protein serine/threonine kinase activity	GO:0071902	0.595	1
response to oxidative stress	GO:0006979	0.596	1
positive regulation of establishment of protein localization	GO:1904951	0.596	1
organic acid metabolic process	GO:0006082	0.598	3
fatty acid metabolic process	GO:0006631	0.598	1
regulation of cell-cell adhesion	GO:0022407	0.598	1
ribonucleoside triphosphate metabolic process	GO:0009199	0.598	1
purine nucleoside triphosphate metabolic process	GO:0009144	0.599	1
vesicle organization	GO:0016050	0.601	1
spermatogenesis	GO:0007283	0.603	1
positive regulation of protein transport	GO:0051222	0.603	1
mitotic cell cycle phase transition	GO:0044772	0.603	1
macromolecule methylation	GO:0043414	0.607	1
response to cytokine	GO:0034097	0.607	2
nucleic acid phosphodiester bond hydrolysis	GO:0090305	0.612	1
chromosome segregation	GO:0007059	0.612	1
positive regulation of cytokine production	GO:0001819	0.613	1
male gamete generation	GO:0048232	0.614	1
positive regulation of catalytic activity	GO:0043085	0.617	4
regulation of protein kinase activity	GO:0045859	0.624	2
positive regulation of cell migration	GO:0030335	0.628	1
protein oligomerization	GO:0051259	0.628	1
coenzyme metabolic process	GO:0006732	0.628	1

anion transmembrane transport	GO:0098656	0.642	1
reproductive structure development	GO:0048608	0.642	1
positive regulation of cell motility	GO:2000147	0.642	1
regulation of system process	GO:0044057	0.643	1
regulation of small GTPase mediated signal transduction	GO:0051056	0.643	1
cell growth	GO:0016049	0.643	1
negative regulation of catalytic activity	GO:0043086	0.643	2
reproductive system development	GO:0061458	0.644	1
nucleobase-containing compound catabolic process	GO:0034655	0.65	1
positive regulation of cellular component movement	GO:0051272	0.651	1
transmembrane transport	GO:0055085	0.652	5
cellular homeostasis	GO:0019725	0.658	2
positive regulation of locomotion	GO:0040017	0.658	1
cellular amino acid metabolic process	GO:0006520	0.663	1
negative regulation of proteolysis	GO:0045861	0.667	1
regulation of kinase activity	GO:0043549	0.667	2
establishment of organelle localization	GO:0051656	0.675	1
cell division	GO:0051301	0.686	1
cellular response to organic cyclic compound	GO:0071407	0.686	1
cellular response to DNA damage stimulus	GO:0006974	0.686	2
methylation	GO:0032259	0.691	1
signal transduction	GO:0007165	0.691	20
regulation of intracellular transport	GO:0032386	0.693	1
aromatic compound catabolic process	GO:0019439	0.694	1
lipid localization	GO:0010876	0.7	1
regulation of cellular protein localization	GO:1903827	0.71	1

signaling	GO:0023052	0.714	21
protein localization to membrane	GO:0072657	0.714	1
cofactor metabolic process	GO:0051186	0.714	1
positive regulation of MAPK cascade	GO:0043410	0.715	1
positive regulation of protein phosphorylation	GO:0001934	0.716	2
defense response to other organism	GO:0098542	0.718	1
negative regulation of intracellular signal transduction	GO:1902532	0.719	1
protein dephosphorylation	GO:0006470	0.726	1
regulation of protein serine/threonine kinase activity	GO:0071900	0.726	1
cytokine-mediated signaling pathway	GO:0019221	0.727	1
single-organism carbohydrate metabolic process	GO:0044723	0.736	1
positive regulation of phosphorylation	GO:0042327	0.737	2
positive regulation of protein kinase activity	GO:0045860	0.741	1
regulation of mitotic cell cycle	GO:0007346	0.742	1
cell communication	GO:0007154	0.743	21
Ras protein signal transduction	GO:0007265	0.751	1
organic anion transport	GO:0015711	0.751	1
regulation of cytokine production	GO:0001817	0.751	1
reproduction	GO:0000003	0.764	2
positive regulation of kinase activity	GO:0033674	0.765	1
lipid biosynthetic process	GO:0008610	0.766	1
reproductive process	GO:0022414	0.776	2
cytokine production	GO:0001816	0.787	1
developmental process involved in reproduction	GO:0003006	0.79	1
vesicle-mediated transport	GO:0016192	0.792	3
DNA repair	GO:0006281	0.794	1

negative regulation of cellular component organization	GO:0051129	0.799	1
endocytosis	GO:0006897	0.812	1
organelle organization	GO:0006996	0.813	9
dephosphorylation	GO:0016311	0.817	1
mitotic cell cycle process	GO:1903047	0.829	1
regulation of MAPK cascade	GO:0043408	0.831	1
cellular response to cytokine stimulus	GO:0071345	0.831	1
regulation of response to external stimulus	GO:0032101	0.834	1
defense response	GO:0006952	0.854	2
regulation of GTPase activity	GO:0043087	0.86	1
anion transport	GO:0006820	0.861	1
MAPK cascade	GO:0000165	0.862	1
small GTPase mediated signal transduction	GO:0007264	0.876	1
signal transduction by protein phosphorylation	GO:0023014	0.878	1
mitotic cell cycle	GO:0000278	0.894	1
carbohydrate metabolic process	GO:0005975	0.899	1
oxidation-reduction process	GO:0055114	0.906	2
system process	GO:0003008	0.931	5
cellular lipid metabolic process	GO:0044255	0.932	1
G-protein coupled receptor signaling pathway	GO:0007186	0.948	4
chromosome organization	GO:0051276	0.969	1
neurological system process	GO:0050877	0.973	3
regulation of hydrolase activity	GO:0051336	0.982	1
sensory perception	GO:0007600	0.985	2
detection of stimulus	GO:0051606	0.996	1
